# A new regression model for overdispersed binomial data accounting for outliers and an excess of zeros

**DOI:** 10.1002/sim.9005

**Published:** 2021-05-07

**Authors:** Roberto Ascari, Sonia Migliorati

**Affiliations:** ^1^ Department of Economics, Management and Statistics University of Milano‐Bicocca Milan Italy

**Keywords:** Bayesian inference, binomial regression, intraclass correlation, mixture models, outliers, overdispersion

## Abstract

Binary outcomes are extremely common in biomedical research. Despite its popularity, binomial regression often fails to model this kind of data accurately due to the overdispersion problem. Many alternatives can be found in the literature, the beta‐binomial (BB) regression model being one of the most popular. The additional parameter of this model enables a better fit to overdispersed data. It also exhibits an attractive interpretation in terms of the intraclass correlation coefficient. Nonetheless, in many real data applications, a single additional parameter cannot handle the entire excess of variability. In this study, we propose a new finite mixture distribution with BB components, namely, the flexible beta‐binomial (FBB), which is characterized by a richer parameterization. This allows us to enhance the variance structure to account for multiple causes of overdispersion while also preserving the intraclass correlation interpretation. The novel regression model, based on the FBB distribution, exploits the flexibility and large variety of the distribution's possible shapes (which includes bimodality and various tail behaviors). Thus, it succeeds in accounting for several (possibly concomitant) sources of overdispersion stemming from the presence of latent groups in the population, outliers, and excessive zero observations. Adopting a Bayesian approach to inference, we perform an intensive simulation study that shows the superiority of the new regression model over that of the existing ones. Its better performance is also confirmed by three applications to real datasets extensively studied in the biomedical literature, namely, bacteria data, atomic bomb radiation data, and control mice data.

## 1 INTRODUCTION

Binary outcomes are common in biomedical research. For example, they occur in bioassay experiments, where the focus is on dose‐response relationships,^1^, ^2^, ^3^ in entomology, where the number of insects that respond to some stimulus is observed,^4^ and in epidemiology, with reference to chromosomally aberrant cells,^5^ cohort studies,^6^ or health‐related evaluations of quality of life.^7^ Indeed they are also encountered in many other similar fields.^8^, ^9^, ^10^ The binomial model is often used for this kind of discrete data,^11^ where the probability of “success” is assumed to remain constant throughout the independent Bernoulli trials leading to the value of the binomial response variable. However, it is possible for the data to be overdispersed, that is, to be characterized by a larger variance than assumed by the model. In practice, this happens almost every time that binomial regression (BinReg) is applied to count data.^12^ However, ignoring overdispersion can lead to a serious underestimation of standard errors, and to misleading inferences.^13^

The phenomenon of overdispersion has been widely addressed in the literature, and the most popular model for overdispersed data is the beta‐binomial (BB) model, proposed originally by Williams^14^ and later applied in many contexts.^2^, ^3^, ^7^, ^15^, ^16^, ^17^, ^18^, ^19^ Several different causes of overdispersion are reviewed in the literature,^4^, ^13^ which are mainly connected with the failure of the basic i.i.d. assumption of the individual responses (ie, the binary outcomes) within a covariate pattern. The BB model accounts for overdispersion, allowing the probability of “success” π to vary according to a beta distribution, thus relaxing the i.i.d. assumption. In particular, the BB model enriches (and encompasses) the binomial model with an additional precision/dispersion parameter, which admits an interesting interpretation in terms of intraclass correlation as well.^16^, ^20^ Nevertheless, there are situations where overdispersion is due to concomitant causes, and a single additional parameter is unable to account for all of them. For example, in teratology experiments, overdispersion is naturally induced by the fact that litters, rather than individual animals, are regarded as the experimental units. Therefore, since we expect differences between litters on biological grounds, the dispersion parameter of the BB model is naturally dedicated to accounting for this source of overdispersion, disregarding any further sources. Of particular relevance are situations where, besides the omission of important explanatory variables, overdispersion is due to (or exaggerated by) the contaminating presence of outliers or excess of zero observations (ie, a sample proportion of zero observations higher than the one assumed by the model).

The purpose of this study is to generalize the BB distribution by introducing the flexible beta‐binomial (FBB) distribution, and to define a new regression model for overdispersed data based on it. The new distribution can be seen as a special mixture of two BB distributions, which displays two further parameters. This allows to enrich the variance structure so as to account for multiple causes of overdispersion, though preserving the intraclass correlation interpretation as well. The great variety of possible shapes of the new FBB distribution (which includes bimodality and various tail behaviors) directly reflects on the flexibility of the corresponding regression model. Indeed, the latter succeeds in adapting to the presence of outliers as well as excessive zero observations without requiring ad hoc extra components accounting for them. This is possible because the new model dedicates one of its mixture components to a particular group of observations (eg, zero‐values and/or outliers) automatically and only when necessary, providing interesting information about the possible sources of overdispersion. We adopt a Bayesian approach to inference, which is more suitable for complex models such as mixtures, as it avoids the computational and analytical problems of likelihood‐based inference and its small‐sample limitations. The potential of the new model is illustrated by means of three datasets extensively studied in the literature, all characterized by nonstandard modeling issues. More precisely, we focus on bacteria data related to a completely randomized experiment aimed at comparing two different biotypes of egg parasitoid, and characterized by a large amount of zero counts.^21^ Then, we analyze atomic bomb radiation data concerning the study of chromosomal abnormalities of cells from survivors of the atomic bombs in Hiroshima and Nagasaki, where some latent groups are present.^5^ Finally, we apply our model to control mice data referring to fetal deaths in groups of mice for different litters.^22^, ^23^ Here it is well recognized the presence of outlying observations. For all the examples, the new model is confirmed to be preferable to competing models in terms of fit via several diagnostics designed to detect discrepancies between observed and predicted data. The remainder of this article is organized as follows: Section 2 briefly describes some useful distributions for binary outcomes and their related regression models and introduces a novel regression model based on the new FBB distribution. Section 3 describes the Bayesian approach to inference as well as several model comparison criteria and model checks based on posterior predictive distributions and cross‐validated leave‐one‐out (loo) approaches. Section 4 outlines the simulation studies used to evaluate the performance of the flexible beta‐binomial regression (FBBReg) model, and compares it with that of the BinReg and the beta‐binomial regression (BBReg) models. Section 5 discusses the results and main findings from the application of our new regression model to real datasets. Finally, Section 6 offers some concluding remarks.

## 2 DISTRIBUTIONS AND REGRESSION MODELS

In this section, we briefly review the BB distribution and introduce the new FBB one. Then, we illustrate the regression models based on these distributions (BBReg and FBBReg, respectively).

### 2.1 The BB distribution

Let Y be a response variable denoting the sum of n independent Bernoulli variables with probability parameter π. Assuming that π is constant leads to the binomial distribution: Y∼Bin(n,π) with probability mass function (pmf) $f_{Bin}(y;\pi )=\left(\begin{array}{c} n\\ y \end{array}\right)\pi^{y}(1-{\pi )}^{n-y}$.

If we sum dependent Bernoulli variables whose probability parameter is random and follows a beta distribution, we obtain a BB distribution. Specifically, given π, we have Y|π∼Bin(n,π), where π∼Beta(μ,ϕ) with probability density function (pdf)

(1) $$f_{Be}(\pi ;\mu ,\phi )=\frac{1}{B(\phi \mu ,\phi (1-\mu ))}\pi^{\phi \mu -1}(1-{\pi )}^{\phi (1-\mu )-1},$$

(0<π<1). Here, 0<μ<1 is the mean 𝔼[π], ϕ>0 represents a precision parameter, $B(a,b)=\frac{\Gamma (a)\Gamma (b)}{\Gamma (a+b)}$ is the Beta function, and Γ(·) is the Gamma function. The pmf $f_{BB}(y;\mu ,\phi )$ of Y can be easily obtained by marginalization:

(2) $$f_{BB}(y;\mu ,\phi )=\left(\begin{array}{c} n\\ y \end{array}\right)\frac{B(\phi \mu +y,\phi (1-\mu )+n-y)}{B(\phi \mu ,\phi (1-\mu ))},$$

where y∈{0,1,...,n} and n∈ℕ. In particular, we have

(3) $$𝔼\left[Y\right]=n\mu ,\;\mathrm{Var}\left(Y\right)=n\mu (1-\mu )\left[1+\frac{\left(n-1\right)}{\phi +1}\right],$$

where Var(Y) is derived by applying the law of total variance. Note that the parameter $\theta =\frac{1}{\phi +1}$ can be thought of as an overdispersion parameter since Var(Y) is an increasing function of θ, and the form of Var(Y) approximates the binomial variance as θ→0. Moreover, θ also admits an interesting interpretation in terms of the intraclass correlation coefficient (ICC),^16^ that is, it represents the (common) correlation between the pairs of the Bernoulli variables that form the response count Y. In particular, let $U_{1},...,U_{n}$ be the Bernoulli variables giving rise to the response count $Y=\sum_{r=1}^{n}U_{r}$, and suppose they are identically distributed (with expected value μ), but not independent. Then, the expression of Var(Y) given by (3) together with the well‐known equality $\mathrm{Var}\left(Y\right)=\sum_{r=1}^{n}\mathrm{Var}\left(U_{r}\right)+2\sum_{r<l}\text{Cov}\left(U_{r},U_{l}\right)$, allow to prove that:

(4) $$\text{Cov}\left(U_{r},U_{l}\right)=\mu (1-\mu )\left[\frac{1}{\phi +1}\right],$$

for r,l=1,...,n,r≠l. Given (4), the ICC takes the form

$$\rho_{BB}=\frac{\text{Cov}\left(U_{r},U_{l}\right)}{\mu (1-\mu )}=\frac{1}{\phi +1}=\theta ,$$

and the variance (3) of the BB can be written as $\mathrm{Var}\left(Y\right)=n\mu (1-\mu )\left(1+(n-1)\rho_{BB}\right).$

### 2.2 The FBB distribution

The FBB distribution is obtained by compounding the binomial distribution with the flexible beta (FB) one. The latter is the univariate case of the flexible Dirichlet (FD) distribution, which is a generalization of the Dirichlet distribution.^24^, ^25^ In particular, the FB distribution is a special mixture of two beta distributions with a common precision parameter ϕ and two arbitrary means $\lambda_{1}>\lambda_{2}$.^26^ Its pdf can be expressed as

$$f_{FB}(\pi ;\lambda_{1},\lambda_{2},\phi ,p)=pf_{Be}(\pi ;\lambda_{1},\phi )+(1-p)f_{Be}(\pi ;\lambda_{2},\phi ),$$

where $f_{Be}(\cdot ;\cdot )$ is given by formula (1), $0<\lambda_{2}<\lambda_{1}<1$, ϕ>0, and 0<p<1 is the mixing proportion. From a regression perspective, a convenient reparameterization of the FB distribution is given by

$$\left\{\begin{array}{c} \mu =p\lambda_{1}+(1-p)\lambda_{2}\\ \phi =\phi \end{array}\right.\left\{\begin{array}{c} w=\frac{\lambda_{1}-\lambda_{2}}{\min\{\mu /p,(1-\mu )/(1-p)\}}\\ p=p \end{array}\right.$$

which explicitly includes the mean 0<μ=𝔼[π]<1 and a normalized distance 0<w<1 between the two mixture components. This parameterization proves to be particularly useful since it defines a variation‐independent parametric space, meaning that no constraints exist among the parameters μ,w,ϕ, and p. Specifically, the mean and variance of the FB distribution are given by

(5) $$𝔼\left[\pi \right]=\mu ,\;\mathrm{Var}\left(\pi \right)=\frac{\mu (1-\mu )}{\phi +1}\left[1+\phi \;w^{2}m(\mu ,p)\right],$$

where

(6) $$m(\mu ,p)=\min\left(\frac{\mu (1-p)}{p(1-\mu )},\frac{(1-\mu )p}{(1-p)\mu }\right).$$

Now, let Y|π∼Bin(n,π) and π∼FB(μ,w,ϕ,p). Then, the compound distribution Y∼FBB(n,μ,w,ϕ,p) has pmf

(7) $$f_{FBB}(y;\mu ,w,\phi ,p)=pf_{BB}(y;\lambda_{1},\phi )+(1-p)f_{BB}(y;\lambda_{2},\phi ),$$

where $f_{BB}(\cdot ;\cdot )$ is given by (2) and

(8) $$\lambda_{1}=\mu +(1-p)\;w\min\left(\frac{\mu }{p},\frac{1-\mu }{1-p}\right),\;\lambda_{2}=\mu -p\;w\min\left(\frac{\mu }{p},\frac{1-\mu }{1-p}\right).$$

**FIGURE 1:**
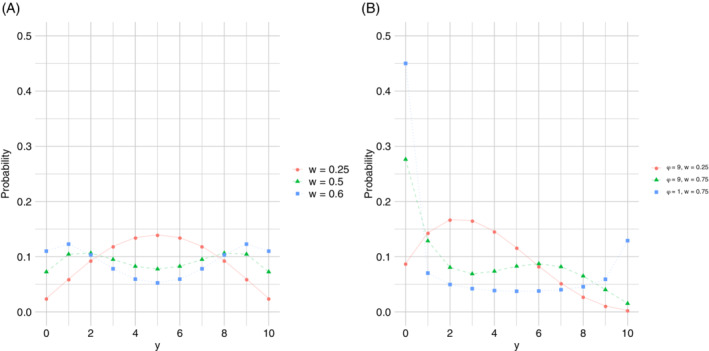
Probability mass function of the FBB distribution with n=10,μ=0.5,p=0.5, and ϕ=9, A, and n=10,μ=1/3, and p=0.5, B[Color figure can be viewed at wileyonlinelibrary.com]

From Equation (7), it is immediately clear that the FBB distribution can be expressed as a finite mixture of two BB components with a common precision parameter ϕ and different means $n\lambda_{1}>n\lambda_{2}$. This allows a large extension of the possible shapes of the FBB distribution compared to those possible with the BB distribution, which is inherited from the considerable variety of shapes of the FB distribution.^26^ In particular, in addition to the usual unimodal shape, J‐shape, inverse J‐shape, and U‐shape that are possible with the BB distribution, the FBB distribution can be bimodal, asymmetric, and can also accommodate for various tail behaviors, as illustrated in Figure 1. In particular, the FBB can exhibit both symmetric unimodal (solid line in A) and bimodal probability functions (dashed and dotted lines in A). Of particular interest are the tail behaviors (B), since the FBB can give rise to unimodal distributions with heavy tails, and it includes pmfs resulting in only one heavy tail, and possibly large asymmetry. Moreover, note that the FBB contains the BB distribution as an inner point. Indeed, fixing μ=p, w=1/ϕ, and ϕ=ν+1 (ν>0) it is possible to show that FBB(n,μ,w,ϕ,p) $\overset{d}{=}$ BB(n,μ,ν).

Equation (5) allows us to compute the mean and variance of the FBB, which take the form

(9) $$𝔼\left[Y\right]=n\mu ,\;\mathrm{Var}\left(Y\right)=n\mu (1-\mu )\left[1+\frac{\left(n-1\right)}{\phi +1}+\frac{\left(n-1\right)}{\phi +1}\phi \;w^{2}\;m(\mu ,p)\right],$$

where m(μ,p) is given by (6). A comparison between the FBB variance (9) and the BB variance (3) indicates that the former includes the extra variation due to overdispersion (second addend enclosed in square brackets) already present in the BB variance, which becomes zero when the parameter $\theta =\frac{1}{\phi +1}\to 0$ (ie, when the precision ϕ of the beta distribution tends to infinity). However, the FBB variance (9) further includes a third addend, which can take on positive values when θ→0, and depends on the normalized distance w between the mixture components and the mixing proportion p, approaching zero if one of the following limits holds: w→0,p→0,orp→1. Therefore, the third addend in (9) is due to the presence of two clusters, typically attributable to an unobserved (or unobservable) qualitative explanatory variable. Very interestingly, we see in Section 4 that one of these two clusters can be adapted to capture a group of outliers or an excess of zero observations where appropriate.

Finally, it is noteworthy that the FBB variance admits an interesting interpretation in terms of intraclass correlation. In particular, taking advantage of the notation used in Section 2.1, the covariance between any two Bernoulli variables forming the count Y can be written as

(10) $$\text{Cov}\left(U_{r},U_{l}\right)=\mu (1-\mu )\left\{\frac{1}{\phi +1}+\frac{\phi }{\phi +1}w^{2}m(\mu ,p)\right\},$$

for r,l=1,...,n,r≠l. Given (10), the ICC of the FBB distribution takes the form

(11) $$\rho_{FBB}=\frac{1}{\phi +1}+\frac{\phi }{\phi +1}w^{2}m(\mu ,p)=\theta +(1-\theta )w^{2}m(\mu ,p),$$

where $\theta =\frac{1}{\phi +1}$. Moreover, the variance (9) of the FBB can be rewritten as $\mathrm{Var}\left(Y\right)=n\mu (1-\mu )\left\{1+(n-1)\rho_{FBB}\right\}.$ Equation (11) shows that $\rho_{FBB}$ can be interpreted as a weighted mean of the maximum possible correlation (ie, 1) and the minimum possible correlation $0<w^{2}m(\mu ,p)<1$ for given θ. Note also that it is an increasing function of w, and it approaches $\theta =\frac{1}{\phi +1}$ (ie, the ICC of the BB distribution) when one of the following conditions holds: w→0,p→0,orp→1, that is, whenever the two latent clusters collapse. Moreover, pandμ enter $\rho_{FBB}$ in a symmetric fashion. Finally, it is noteworthy that, differently from the BB intraclass correlation, the FBB one does not depend on ϕ only but also on μ, which means that, in a regression context, it is naturally modeled as a function of covariates.

A further investigation of the behavior of the FBB's ICC can be found in Section 2.1 of the Supplementary Material (SM).

### 2.3 Excess of zeros

Binomial data are often affected by an excess of zeros, that is, a larger proportion of zero values than the one allowed by the assumed model. Let $\delta_{0}^{f}$ be the probability of the event “zero successes among *n* trials” for distribution f. Then, for a BB distribution, we have

(12) $$\delta_{0}^{BB(\mu ,\phi )}=\frac{\Gamma \left(\phi (1-\mu )+n\right)\Gamma \left(\phi \right)}{\Gamma \left(\phi +n\right)\Gamma \left(\phi (1-\mu )\right)}=\frac{{\left(\phi (1-\mu )\right)}^{\left[n\right]}}{\phi^{\left[n\right]}},$$

where *x* ^[*n*]^ = *x*(*x* + 1) … (*x* + *n* − 1) is the rising factorial function. Indeed, the inability of the BB to accommodate excessive zero counts is mainly due to the limited range of its tail behaviors. In contrast, the extreme flexibility of the FBB distribution in terms of shapes proves beneficial for modeling zero counts. In particular, from (7) and (12), it follows that

(13) $$\begin{array}{ll} \delta_{0}^{FBB(\mu ,w,\phi ,p)} & =p\;\delta_{0}^{BB(\lambda_{1},\phi )}+(1-p)\;\delta_{0}^{BB(\lambda_{2},\phi )}\\ & =\frac{p\;{\left(\phi (1-\lambda_{1})\right)}^{\left[n\right]}+(1-p)\;{\left(\phi (1-\lambda_{2})\right)}^{\left[n\right]}}{\phi^{\left[n\right]}}, \end{array}$$

where $\lambda_{1}$ and $\lambda_{2}$ are given by (8). From an analytical point of view, one observes that $\delta_{0}^{FBB(\mu ,w,\phi ,p)}$ is an increasing function of w, tending to $\delta_{0}^{BB(\mu ,\phi )}$ when w→0. This happens because both $\lambda_{1}$ and $\lambda_{2}$ collapse to μ and, therefore, the two mixture components coincide (please note that w=0 does not belong to the parameter space). Analogously, $\delta_{0}^{FBB(\mu ,w,\phi ,p)}\to \delta_{0}^{BB(\mu ,\phi )}$ when p goes to the boundary of its parameter space, as it easily follows from (13) once the following limits are taken into account:

$$\left\{\begin{array}{c} \lim_{p\to 0}\;\lambda_{1}=\mu (1-w)+w\\ \lim_{p\to 0}\;\lambda_{2}=\mu \end{array}\right.\left\{\begin{array}{c} \lim_{p\to 1}\;\lambda_{1}=\mu \\ \lim_{p\to 1}\;\lambda_{2}=\mu (1-w). \end{array}\right.$$

In addition, $\delta_{0}^{FBB(\mu ,w,\phi ,p)}$ takes its maximum value when p=μ. It is noteworthy that, for fixed μ, the probability of zero values decreases as ϕ increases. Conversely, for fixed ϕ, as μ approaches 1, the probability of zero values tends to 0. This is a reasonable result since if the overall mean approaches its upper limit, most of the probability mass should be located in a neighborhood of 1. A graphical inspection of the behavior of $\delta_{0}^{FBB(\mu ,w,\phi ,p)}$ can be found in Section 2.2 of the SM.

Note that a zero‐inflated binomial (or BB) model^15^, ^27^, ^28^ is a two (respectively three) parameters model that accounts for the excess of zeros by expressly dedicating an ad hoc parameter to the zero‐inflation. In particular, let Y be a random variable distributed according to a zero‐inflated binomial (ZIBin) or to a zero‐inflated BB (ZIBB) distribution with inflation parameter q. Then, its pmf can be expressed as

$$f_{ZI}(y;q,\cdot )=\left\{\begin{array}{cc} q+(1-q)f(0;\cdot ),\; & \text{if}\;y=0,\\ (1-q)f(y;\cdot ),\; & \text{if}\;y\in \{1,2,...,n\}, \end{array}\right.$$

where f(y;·) is the pmf of the proper binomial or BB distribution.

Note that, differently from inflated models, the FBB succeeds in addressing this issue by dedicating a component of its mixture to zero inflation automatically, and only when necessary, as we see in Section 4.

Almost all the properties of the FBB distribution are due to its mixture expression, which is inherited from the FB distribution. Indeed, the latter is a structured (ie, non generic) mixture with constraints on its components' parameters ensuring model identifiability. Despite these models are characterized by only two mixture components, they are flexible enough to handle several issues that occur quite often in applications, though preserving good theoretical properties (differently from generic mixtures).

### 2.4 The BinReg, BBReg, and FBBReg models

Let $Y_{i}$ represent the response variable observed for subject i(i=1,...,N), that is, the count of successes out of a sample of size $n_{i}$ and let $𝔼\left[Y_{i}\right]=n_{i}\mu_{i}$. Furthermore, let ${\text{x}}_{i}={\left(1,x_{i1},\ldots ,x_{iK}\right)}^{⊺}$ be a (*K* + 1)‐dimensional vector of covariates for subject i. Then, it is possible to link the mean parameter $\mu_{i}=𝔼\left[Y_{i}/n_{i}\right]$ to the linear predictor by following the GLM methodology^11^

(14) $$g(\mu_{i})={\text{x}}_{i}^{⊺}\beta ,\;i=1,\ldots ,N,$$

where $\beta ={\left(\beta_{0},\beta_{1},\ldots ,\beta_{K}\right)}^{⊺}$ is a vector of regression coefficients, and g(·) is a twice differentiable and strictly monotone link function. Given that $\mu_{i}$ takes values in the unit interval (0, 1), a straightforward choice for g(·) is $\log\text{it}(\mu_{i})=\log(\mu_{i}/(1-\mu_{i}))$. Although other link functions can be adopted (eg, the probit or the complementary log‐log), the logit link is a popular choice since it is the canonical link function for the binomial distribution, also allowing a simple interpretation in terms of odds ratios as well. The BinReg, BBReg, and FBBReg models are then defined by assuming that $Y_{i}$ follows a $\mathrm{Bin}(n_{i},\pi_{i})$, $\text{BB}(n_{i},\mu_{i},\phi )$, or $\text{FBB}(n_{i},\mu_{i},w,\phi ,p)$ distribution, respectively. In the case of the BinReg, the parameter $\mu_{i}$ in Equation (14) must be replaced with $\pi_{i}$. The inflated ZIBin and ZIBB regression models (ZIBinReg and ZIBBReg, respectively) can be defined in a similar way.

## 3 ESTIMATION ISSUES

None of the three regression models described in Section 2.4 admits an explicit solution to the estimation problem. Moreover, neither the BB nor the FBB distribution belongs to the dispersion exponential family; thus, and differently from the binomial, the estimation of their parameters cannot be conducted by simply applying the standard iteratively reweighted least squares method.^11^ There are many proposals in the literature for how to address the issues in likelihood‐based inference within BB, as well as BBReg, models.^20^, ^29^, ^30^, ^31^

We decided to adopt a Bayesian approach, which is particularly convenient for dealing with complex models such as mixtures, or simply with models involving many parameters. Moreover, this approach does not depend on asymptotic calculations and makes it easy to cope with the small sample problems that typically affect maximum likelihood inference.

Since the FBB is a finite mixture (see Equation (7)), it can always be expressed as an incomplete data model where the allocation of each observation to one of the mixture components is unknown. Therefore, a Bayesian approach based on Markov chain Monte Carlo (MCMC) techniques is particularly suitable, producing posterior (simulated) distributions for the parameter vector. In particular, we take advantage of the Hamiltonian Monte Carlo (HMC) algorithm,^32^, ^33^ which generalizes one of the most well‐known MCMC, namely the Metropolis algorithm, by combining MCMC and deterministic simulation methods to generate efficient transitions. This is achieved also by considering the derivatives of the pdf of the target distribution (ie, the posterior). The popularity of HMC is increasing because it is more efficient than classical MCMC methods, and because it is easy to perform through the Stan modeling language which uses the standard No‐U‐Turn Sampler.^34^ Its implementation requires the specification of the log‐likelihood function and prior distributions for the parameters. Let y be an i.i.d. sample of size N from the response Y. Then, the log‐likelihood is

$$l(\eta |\text{y})=\sum_{i=1}^{N}\log\left(f^{*}(y_{i};\eta )\right),$$

where $f^{*}(\cdot ;\cdot )$ denotes the pmf of the assumed distribution (binomial, BB, or FBB), and η is its parameter vector. In the special case of the FBBReg, $f^{*}(\cdot ;\cdot )$ is given by Equation (7) and $\eta ={\left(\beta ,w,\phi ,p\right)}^{⊺}$.

As for the priors, the variation‐independent parameter space allows us to assume prior independence, which is the usual choice when no prior information is available. Thus, we can specify a prior distribution for each parameter separately. In the rest of this article, we use a diffuse multivariate normal prior for the regression coefficients, that is, $\beta ∼N_{K+1}(\text{0};\sum )$, where **0** is the zero vector and ∑ is a diagonal covariance matrix with large values for the variances. Furthermore, we adopt a uniform distribution on (0, 1) for $\theta =\frac{1}{\phi +1},w,\text{and}\;p.$ Please note that BinReg requires only the specification of the prior for β; the BBReg also involves ϕ whereas the FBBReg requires the specification of all the four priors. These choices represent a non‐ (or weakly) informative—but still proper—option. Naturally, different priors can be considered, for example, a widespread method is a Gamma(k·g,g) for ϕ, with small values of g>0 to induce a large variability around the prior mean k>0. However, we prefer the uniform prior for $\theta =\frac{1}{\phi +1}$ since it guarantees non‐informativeness without requiring the specification of hyperparameters. A sensitivity study concerning priors has highlighted robustness, understood as a limited impact on inferential conclusions (see Section 1 of the SM).

Both in the simulations and real data applications, we diagnose convergence of the chains to the equilibrium distribution through graphical tools (trace and density plots), Geweke and Heidel diagnostics to ascertain stationarity, as well as through potential scale reduction and effective sample size to ascertain mixing of the chains.^35^ To diminish the dependence of the results on the starting values, we choose these values randomly, and we discard the first half of each chain, imposing a warm‐up of 50%. We do not need to set thinning intervals different from 1 (ie, we keep every element from each chain without discarding some) due to the large effective sample sizes produced by the HMC and to the low level of autocorrelation.

### 3.1 Bayesian diagnostic

To compare the three considered regression models, we use a fully Bayesian goodness‐of‐fit index, namely, the Watanabe‐Akaike information criterion (WAIC),^36^, ^37^ which is well‐defined also for non‐regular models such as mixtures. WAIC uses the log pointwise posterior predictive density (lppd) as a measure of fit, and the effective number of parameters ${\hat{p}}_{\text{WAIC}}$ as a correction for the model's complexity. Given a sample of size B ${\left(\eta^{\left(1\right)},\eta^{\left(2\right)},\ldots ,\eta^{\left(B\right)}\right)}^{⊺}$ simulated from the posterior distribution, we can compute these quantities as

$$\hat{\text{lppd}}=\overset{N}{\underset{i=1}{\sum }}\log\left(\frac{1}{B}\overset{B}{\underset{b=1}{\sum }}\;f^{*}\left(y_{i}|\eta^{\left(b\right)}\right)\right),$$

$${\hat{p}}_{\text{WAIC}}=\overset{N}{\underset{i=1}{\sum }}\;V_{b=1}^{B}\left(\log f^{*}\left(y_{i}|\eta^{\left(b\right)}\right)\right),$$

where $V_{b=1}^{B}(q_{b})$ denotes the sample variance of the vector ${\left(q_{1},\ldots ,q_{B}\right)}^{⊺}$. Finally, WAIC is defined on the deviance scale, that is $\text{WAIC}=-2\left(\hat{\text{lppd}}-{\hat{p}}_{\text{WAIC}}\right)$. Recently, Vehtari et al^37^ proposed an efficient way to compute a more robust loo cross‐validation criterion. In all our results, the WAIC and loo indexes are very close to each other. Thus, we report only WAIC, which is so far the most widespread one.

Another popular Bayesian diagnostic tool is posterior predictive checks, which aim to assess the validity of a model's assumptions. The main idea of this technique is that “replicated” data generated under the fitted model should behave similarly to the observed data; any differences between the simulated and observed data suggest a potential lack of fit for the model. Let $\eta^{\left(b\right)}(b=1,...,B)$ be an element of a sample simulated from the posterior distribution, and let $y^{\left(b\right)}$ be a sample generated from the posterior predictive distribution $f_{Y}\left(\text{y}|\eta^{\left(b\right)}\right)$. Furthermore, let T(·) be a function of data and model parameters many authors refer to as a *discrepancy measure*.^38^, ^39^ Then, it is possible to compare the empirical distribution of $T\left(y^{\left(b\right)}\right)(b=1,...,B)$ with that of T(y) (ie, the value of T(·) computed based on the observed data). Such a comparison can be conducted via plots or through posterior predictive *p*‐values defined as $P\left(T\left({\text{y}}^{\left(b\right)}\right)\geq T(\text{y})|\text{y}\right)$ (the closer to 0.5, the better). Posterior predictive checks are particularly useful for detecting overdispersion in a Bayesian framework, where classical tools based on deviance and/or Person's $\chi^{2}$ are not suitable. Indeed, choosing the variance as a discrepancy measure, we can assess how the observed variances behave with respect to the theoretical ones. If the observed variance is far from that of the replicated datasets, we can conclude that the assumed distribution is not suitable for modeling the data.

Another critical issue regarding model diagnostics is outlier detection. Our Bayesian perspective has prompted us to use a tool recently introduced in the literature, namely, the conditional predictive ordinate (CPO).^39^, ^40^, ^41^ This is a measure used to detect unlikely observations given the current model, and it is defined as the predictive density of the *i*th observation once the latter has been excluded from the dataset:

$${\text{CPO}}_{i}=f\left(y_{i}|{\text{y}}_{\left(-i\right)}\right)={\left(\int_{}^{}\frac{1}{f(y_{i}|{\text{y}}_{\left(-i\right)},\eta )}\pi (\eta |\text{y})d\eta \right)}^{-1}.$$

Once a sample of size B has been generated from the posterior distribution of η, and assuming that the $Y_{i}\text{'s}$ are conditionally independent given η, it is possible to obtain an estimate of the CPO:^42^

(15) $${\hat{\text{CPO}}}_{i}=B{\left(\sum_{b=1}^{B}\frac{1}{f\left(y_{i}|\eta^{\left(b\right)}\right)}\right)}^{-1},$$

where $f(y_{i}|\eta^{\left(b\right)})$ is the pmf of the corresponding model with $\eta =\eta^{\left(b\right)}$. Equation (15) estimates CPO_*i*_ as the harmonic mean of the likelihood of $y_{i}$ over all the generated $\eta^{\left(1\right)},\ldots ,\eta^{\left(B\right)}$. Note that this formula allows us to compute an estimate of CPO_*i*_ without fitting the model N times, which would be a very time‐consuming procedure, even for small datasets. The smaller ${\hat{\text{CPO}}}_{i}$ is, the lower the likelihood of observing the ith response given the model so the presence of many small $\hat{\text{CPO}}$ values suggests that the model is not reliable. In Sections 4.3 and 5.3, we use the CPO measure to compare the outlier detection ability of the BinReg, BBReg, and FBBReg models.

## 4 SIMULATION STUDIES

To understand the FBBReg model better, we conduct some simulation studies with different purposes. The first study compares the fitting abilities of the BinReg, BBReg, and the FBBReg models in different data generating processes. Two further studies compare the three regression models in situations that are often problematic for binomial data, namely, the presence of an excess of zeros and the presence of outliers. In each simulation, we estimate the models as described in Section 3, running chains of length 10 000 with a warm‐up of 50%.

### 4.1 Model fit study

To compare the fitting abilities of the models, we consider four scenarios with data generated based on (1) a BBReg, (2) an FBBReg, (3) a mixture of two BBReg's with different means and precision parameters (which is not an FBBReg), and (4) another generic mixture of BBReg's where one precision parameter is very small. Note that in scenarios (1) and (2), the data are generated from two of the three competing models. Thus, here, we can compare the performance of one model when the other is favored, and the performance of the estimation process can be investigated as well. On the other hand, scenarios (3) and (4) inspect more challenging cases where all models are misspecified.

Please note that scenario (4) is affected by two potential causes of overdispersion, namely, the presence of two latent groups (ie, the two mixture components), each one characterized by a different ICC, and an excess of zeros due to the low precision of one component. This is illustrated by Figure S6 in Section 2.3 of the SM, which shows one randomly selected replication from this scenario. For each scenario, we simulate 1000 times a sample of size N=150. More specifically, we generate a single covariate x from a uniform distribution on the interval (−1, 1) and $\text{n}={\left(n_{1},\ldots ,n_{N}\right)}^{⊺}$ as i.i.d. observations from a Poisson distribution with mean parameter equal to 200. A logit link function is adopted to link the mean parameter to the covariate as follows: $\log\text{it}(\mu_{i})=\beta_{0}+\beta_{1}x_{i}$ (i=1,...,N), for fixed $\beta_{0}$ and $\beta_{1}$.

Table 1 shows the biases, the mean square errors (MSEs), and the coverage probabilities resulting from having estimated the parameters through their posterior mean. The last column of Table 1 presents the mean of the WAIC criterion over the 1000 replications. The performance of the FBBReg is better than that of the BinReg in all four scenarios with overdispersion. When overdispersion is due to a common correlation among the binary outcomes forming the binomial count (ie, first scenario with data generated from a BBReg), then the FBBReg is competitively similar to the BBReg, exhibiting similar biases, MSEs, coverage probabilities as well as means of the WAIC criterion. When the presence of a common intraclass correlation cannot explain all the extra variation (second through fourth scenarios), the FBBReg is clearly the best model. Indeed, in the second and third scenarios, the biases and MSEs of $\beta_{0}$ and $\beta_{1}$ under the BBReg model are higher than those of the FBBReg model, and even higher than those of the BinReg model. Moreover, the lowest WAIC values are attained by the FBBReg model. In the fourth scenario, despite the high biases characterizing both the BBReg and the FBBReg, the latter shows a substantial better fit, being the preferable model in 100% of replications.

**TABLE 1:** Model fit simulation study: Bias, MSE (in parentheses), and coverage level (bold) of the parameter estimates. Last column shows the mean values of WAIC criterion (% of times the FBBReg model was selected over the BinReg and BBReg models is provided in parentheses)

Scen.	Model	$\beta_{0}$	$\beta_{1}$	ϕ	p	w	WAIC
(1)	True	1	3	100	(–)	(–)	(–)
	BinReg	0.0006 (0.001)	0.004 (0.004)	(–)	(–)	(–)	1182.68 (100%)
		**0.748**	**0.724**				
	BBReg	−0.0001 (0.001)	0.003 (0.004)	2.267 (326.920)	(–)	(–)	1052.35 (11.7%)
		**0.957**	**0.927**	**0.964**			
	FFBReg	−0.001 (0.001)	−0.005 (0.004)	(–)	(–)	(–)	1053.56 (–)
		**0.963**	**0.951**				
(2)	True	1	3	2.333	0.5	0.75
	BinReg	0.005 (0.027)	0.028 (0.095)	(–)	(–)	(–)	12667.154 (100%)
		**0.144**	**0.172**				
	BBReg	−0.120 (0.032)	−0.244 (0.1225)	(–)	(–)	(–)	1088.75 (96.81%)
		**0.830**	**0.851**				
	FBBReg	−0.0113 (0.019)	0.0078 (0.073)	−0.088 (0.349)	−0.007 (0.003)	−0.074 (0.018)	1078.40 (–)
		**0.948**	**0.950**	**0.925**	**0.973**	**0.958**	
(3)	True	1	3	$\phi_{1}=10$, $\phi_{2}=20$	0.5	(–)
	BinReg	−0.0005 (0.011)	0.018 (0.038)	(–)	(–)	(–)	7178.75 (100%)
		**0.262**	**0.269**				
	BBReg	−0.125 (0.023)	−0.380 (0.172)	(–)	(–)	(–)	1296.14 (100%)
		**0.771**	**0.503**				
	FBBReg	0.045 (0.009)	−0.012 ( 0.023)	(–)	0.001 (0.001)	(–)	1254.96 (–)
		**0.941**	**0.946**		**0.958**	(–)	
(4)	True	1	3	$\phi_{1}=10$, $\phi_{2}=1$	0.8	(–)
	BinReg	0.027 (0.026)	0.0638 (0.077)	(–)	(–)	(–)	7746.50 (100%)
		**0.172**	**0.195**				
	BBReg	−0.292 (0.108)	−0.4835 (0.292)	(–)	(–)	(–)	1299.36 (100%)
		**0.227**	**0.323**				
	FBBReg	−0.274 (0.137)	−0.548 (0.360)	(–)	0.026 (0.0070)	(–)	1192.23 (–)
		**0.36**	**0.151**		**0.606**	(–)	

All these observations suggest using the FBBReg model instead of the BBReg one in general, since the former can recognize a wider spectrum of overdispersion scenarios than the latter, and it does not perform significantly worse when data are generated from a BBReg model. Moreover, the FBBReg yields unbiased estimates even in some scenarios of model misspecification.

### 4.2 Excess of zeros study

In this section, we focus on the performance of the three models in scenarios with a higher percentage of zeros than the one assumed by the binomial data generating process. More precisely, we generate samples of size N=100 such that $Y_{i}∼\mathrm{Bin}\left(n_{i},{\text{logit}}^{-1}\left(1+2x_{i}\right)\right)$, where the number of trials for each observation $n_{i}$ is generated from a Poisson distribution with mean parameter equal to 50, and the continuous covariate x is distributed according to a uniform distribution on the interval (−1.5, 2). Then, in each scenario, we randomly select a different percentage of units (5%, 10%, 20%, and 50%) and set their outcome $Y_{i}$ to zero. For each scenario, we simulate 250 samples. For comparison purposes, we also estimate the zero‐inflated ZIBinReg and ZIBBReg models (see Section 2.3).

**FIGURE 2:**
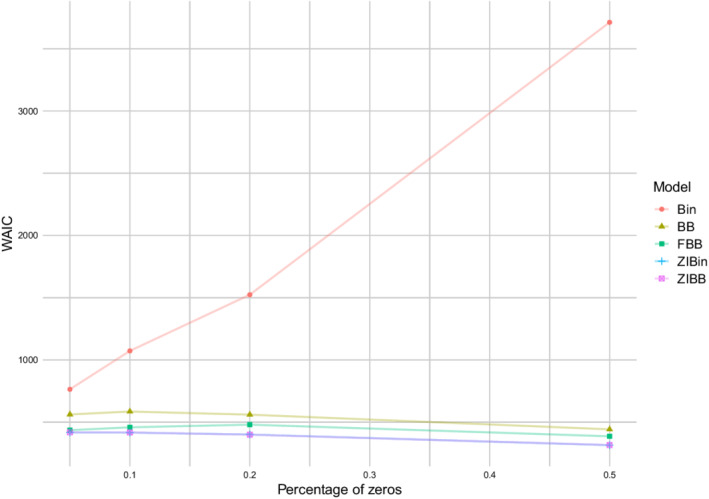
Excess of zeros simulation study: Means of WAICs by model and scenario[Color figure can be viewed at wileyonlinelibrary.com]

Figure 2 reports the mean of WAIC as a function of the percentage of zeros for all models. It is remarkable how the FBBReg performs better than the BinReg and the BBReg models in all scenarios. Moreover, it exhibits WAIC values only slightly worse than both inflated models, which are expressly conceived to handle excess of zero counts. This can be ascribed to the fact that one mixture component of the FBBReg model is dedicated to a particular (even small) group of zero observations. Due to the constraint $\lambda_{1}>\lambda_{2}$ for the component means, the second component is the one devoted to modeling the group of zeros, meaning that its mixing weight is 1‐p. These observations are confirmed by the posterior means of the parameter p (ie, 0.9905, 0.9790, 0.7822, and 0.4684), which are close to the percentage of unchanged observations (ie, 95%, 90%, 80%, and 50%, respectively). Additional details on the posterior distribution of p can be found in Section 3.1 of the SM.

**FIGURE 3:**
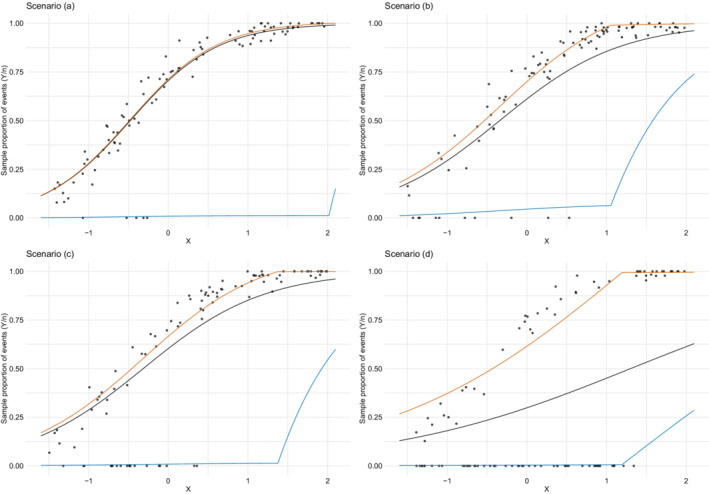
Excess of zeros simulation study: One randomly selected replication for each percentage (5%, 10%, 20%, and 50%) of zero outcomes, and estimated FBBReg curves: μ (black), $\lambda_{1}$ (orange), and $\lambda_{2}$ (blue)[Color figure can be viewed at wileyonlinelibrary.com]

The role of the two mixture components can be better understood by observing that the relationship between the (overall) mean of the response variable μ and the component means $\lambda_{1}$ and $\lambda_{2}$ (see Equation (8)) gives rise to the regression models for $\lambda_{1}$ and $\lambda_{2}$ if μ is considered to be a function of covariates (according to Equation (14)). All the relevant regression curves are reported in Figure 3, which presents a randomly selected replication for each scenario. It is noteworthy that the $\lambda_{2}$ curve almost perfectly adapts to the “zero” group.

### 4.3 Outlier contamination study

**FIGURE 4:**
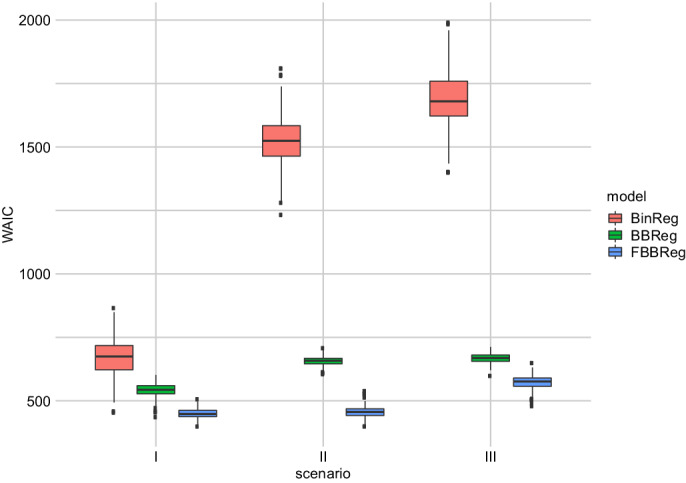
Outlier contamination simulation study: Distribution of WAICs by model and scenario[Color figure can be viewed at wileyonlinelibrary.com]

**FIGURE 5:**
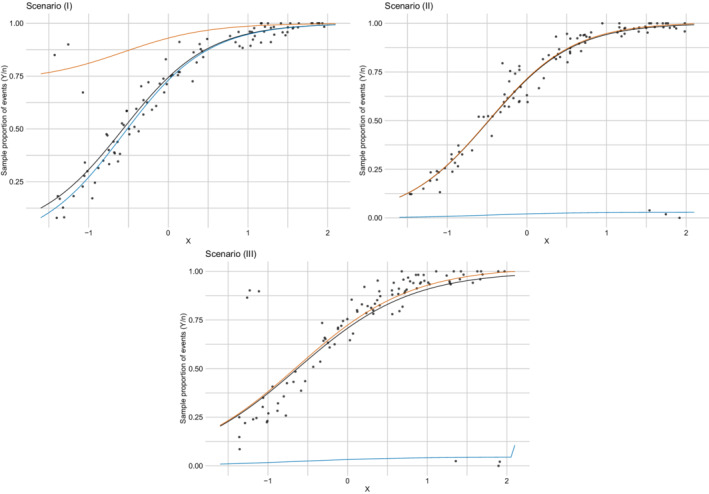
Outlier contamination simulation study: Scatter plot of one randomly selected replication for each outlier contamination scenario, and estimated regression curves for μ (black), $\lambda_{1}$ (orange), and $\lambda_{2}$ (blue)[Color figure can be viewed at wileyonlinelibrary.com]

The last simulation study compares the models in different scenarios with outliers. As in Section 4.2, for each replication, we generate N=100 observations from a $\mathrm{Bin}\left(n_{i},{\text{logit}}^{-1}\left(1+2x_{i}\right)\right)$. Then, we artificially modify the binomial count of a randomly selected subset of observations as $y_{r}^{New}=n_{r}-y_{r}^{Old}$. To ensure that this approach leads to outliers, we have to draw observations corresponding to indexes r from the tails. For this reason, we randomly select three observations (3%) with $x<x_{0.15}$ (scenario I), three observations with $x>x_{0.85}$ (scenario II), and three observations with $x<x_{0.15}$ and three with $x>x_{0.85}$ (scenario III), where $x_{q}$ represents the qth empirical percentile, 0<q<1. Comparing the distribution of the WAIC under the three models in each scenario (Figure 4), we can indicate that BinReg model is the worst in handling outliers, even if the real data generating process is the BinReg itself for the major part of the data. The best model among the considered ones is clearly the FBBReg model. To better understand the reasons for this, consider Figure 5, which shows the scatter plot of one randomly chosen replication together with the estimated regression curves of the FBBReg model for each scenario. One component is entirely dedicated to model a group of outliers. When all the outliers are above (or below) the main cloud of data points (scenarios (I) and (II)), the other component is devoted to modeling the major part of the data. Otherwise, if one group of outliers is placed above the main cloud and one group below it (scenario (III)), only the “most extreme” group is modeled through a mixture component. This is a weakness of the model, which, however, does not undermine its superiority with respect to competing models. Panel (III) of Figure 5 helps to visualize this aspect: the FBBReg model treats the upper‐left group of outliers as units of the same subpopulation of non‐outlying observations (modeled by $\lambda_{1}$) whereas $\lambda_{2}$ models the bottom‐right outliers. All the observations above are also confirmed by the posterior means and CS's of parameter p, reported in Section 3.2 of the SM.

**FIGURE 6:**
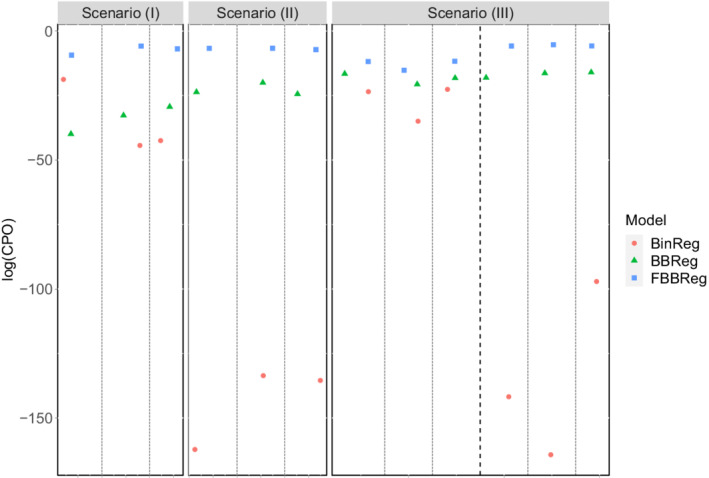
Outlier contamination simulation study: log(CPO)'s of artificially generated outliers. Statistical units are separated by a vertical line; the dashed dark line in panel III divides upper (left) and lower (right) outliers[Color figure can be viewed at wileyonlinelibrary.com]

Finally, we compute the $\hat{\text{CPO}}$ values (see Equation (15)) for the outlying observations in the replications presented in Figure 5. We compare them in Figure 6, where models are represented by shapes. It is easy to see that the FBBReg model leads to higher $\hat{\text{CPO}}$ values in each scenario, confirming its ability to model outliers in a more reliable way. Interestingly, the FBBReg model results in the highest $\hat{\text{CPO}}$ values even for those observations in scenario (III) that are not modeled by a specific mixture component.

## 5 REAL DATA APPLICATIONS

In this section, we analyze three well‐known biomedical datasets, showing how the FBBReg can be used in real data analysis. We compare the FBBReg with the BinReg, BBReg, and with their zero‐inflated versions, if appropriate. Posterior predictive checks and *p*‐values are available in the SM.

### 5.1 Bacteria data (excess of zeros)

Pests can easily infest crops of any kind. To control pests without damaging ecosystems, one can introduce pests' natural enemies in the environment. The *Trichogramma galloi* is an egg parasitoid able to control pests in sugar cane cultivations. Demétrio et al^21^ conducted a completely randomized experiment to compare two different *T. galloi* biotypes, namely, “AA” and “DA.” During the experiment, parasitoid groups with different numbers of female parasitoids were allowed to attempt to parasitize 128 eggs of an alternative host (“Anagasta kuehniella”). There were 10 replicates for each combination of biotype and number of females. The dataset contains the number Y of parasitized eggs (out of $n_{i}=128$) for the “DA” group as well as the number of females. In this dataset, there is a large number of zero counts (namely 37 out of 70, ie, 52.86% of replicates) for each number of females. To estimate the parameters of the regression models, we run four chains of length 20 000 with a warm‐up of 10 000 iterations. Moreover, besides our FBBReg model and the two competitors BinReg and BBReg, we also estimated the zero‐inflated counterparts of the latter two. Table 2A reports the posterior means and 95% credible sets (CS's) of all the parameters of the five models, together with WAIC values, treating the number of females as a numeric variable. More specifically, we defined the linear predictor as a quadratic function of the standardized number of females $(x_{i}),$ that is

$$g(\mu_{i})=\beta_{0}+\beta_{1}x_{i}+\beta_{2}x_{i}^{2},\;i=1,\ldots ,N$$

since there is evidence of presence of a nonlinear relationship.

**TABLE 2:** Bacteria data: Posterior means and 95% CS's for the parameters under the BinReg, BBReg, FBBReg, zero‐inflated BinReg, and zero‐inflated BBReg models, treating the covariate “female” as a quantitative variable, A and as a factor, B

	(A)“Number of female” as quantitative				
Param.	BinReg	BBReg	FBBReg	ZIBinReg	ZIBBReg
$\beta_{0}$	−**0.984**	−**1.688**	−**1.000**	**0.593**	**0.591**
	**(**−**1.058,** −**0.910)**	**(**−**2.374,** −**1.042)**	**(**−**1.457,** −**0.567)**	**(0.485, 0.704)**	**(0.041, 1.162)**
$\beta_{1}$	**0.823**	0.221	**0.687**	**1.393**	**1.530**
	**(0.726, 0.920)**	(−0.527, 0.957)	**(0.268, 1.130)**	**(1.259, 1.531)**	**(0.819, 2.298)**
$\beta_{2}$	−**0.394**	−0.021	−**0.413**	−**0.821**	−**0.891**
	**(**−**0.455,** −**0.333)**	(−0.508, 0.479)	**(**−**0.671,** −**0.161)**	**(**−**0.909,** −**0.737)**	**(**−**1.368,** −**0.437)**
w	(–)	(–)	0.979	(–)	(–)
			(0.936, 0.999)		
ϕ	(–)	0.769	5.216	(–)	4.827
		(0.481, 1.142)	(2.550, 9.347)		(2.698, 7.664)
p	(–)	(–)	0.453	(–)	(–)
			(0.330, 0.576)		
q	(–)	(–)	(–)	0.528	0.523
				(0.413, 0.641)	(0.408, 0.637)
WAIC	4613.8	446.5	408.7	1014.5	405.6
	**(B)“Number of female” as factor**				
**Param.**	**BinReg**	**BBReg**	**FBBReg**	**ZIBinReg**	**ZIBBReg**
$\beta_{0}$	−**1.393**	−**1.677**	−**1.392**	−**0.411**	−0.405
	**(**−**1.532,** −**1.256)**	**(**−**2.823,** −**0.674)**	**(**−**1.974,** −**0.859)**	**(**−**0.572,** −**0.253)**	(−1.015, 0.172)
$\beta_{F4}$	−**3.505**	−1.084	−**2.955**	−**3.387**	−**3.271**
	**(**−**4.210,** −**2.889)**	(−2.809, 0.544)	**(**−**4.579,** −**1.517)**	**(**−**4.227,** −**2.685)**	**(**−**4.918,** −**1.774)**
$\beta_{F8}$	−**0.261**	−0.056	−0.279	−**0.335**	−0.360
	**(**−**0.461,** −**0.058)**	(−1.526, 1.425)	(−1.000, 0.419)	**(**−**0.567,** −**0.103)**	(−1.222, 0.491)
$\beta_{F16}$	−0.080	−0.011	−0.069	−0.108	−0.091
	(−0.277, 0115)	(−1.495, 1.477)	(−0.730, 0.596)	(−0.333, 0.115)	(−0.935, 0.753)
$\beta_{F32}$	**0.925**	0.277	**0.912**	**1.623**	**1.615**
	**(0.746, 1.102)**	(−1.209, 1.787)	**(0.392, 1.480)**	**(1.381, 1.870)**	**(0.736, 2.534)**
$\beta_{F64}$	**0.367**	−0.260	**0.672**	**1.077**	**1.087**
	**(0.182, 0.551)**	(−1.845, 1.286)	**(0.088, 1.275)**	**(0.838, 1.318)**	**(0.201, 2.019)**
$\beta_{F128}$	**0.430**	0.397	0.177	**0.255**	0.222
	**(0.244, 0.613)**	(−1.011, 1.843)	(−0.420, 0.798)	**(0.042, 0.467)**	(−0.562, 1.020)
w	(–)	(–)	0.994	(–)	(–)
			(0.977, 1.000)		
ϕ	(–)	0.793	9.626	(–)	10.263
		(0.495, 1.180)	(5.003, 16.232)		(5.428, 17.076)
p	(–)	(–)	0.511	(–)	(–)
			(0.387, 0.634)		
q	(–)	(–)	(–)	0.521	0.496
				(0.403, 0.637)	(0.374, 0.617)
WAIC	4283.5	454.0	385.0	670.5	382.8

*Note*: Regression coefficients in bold are related to 95% CS's not containing the zero value.

**FIGURE 7:**
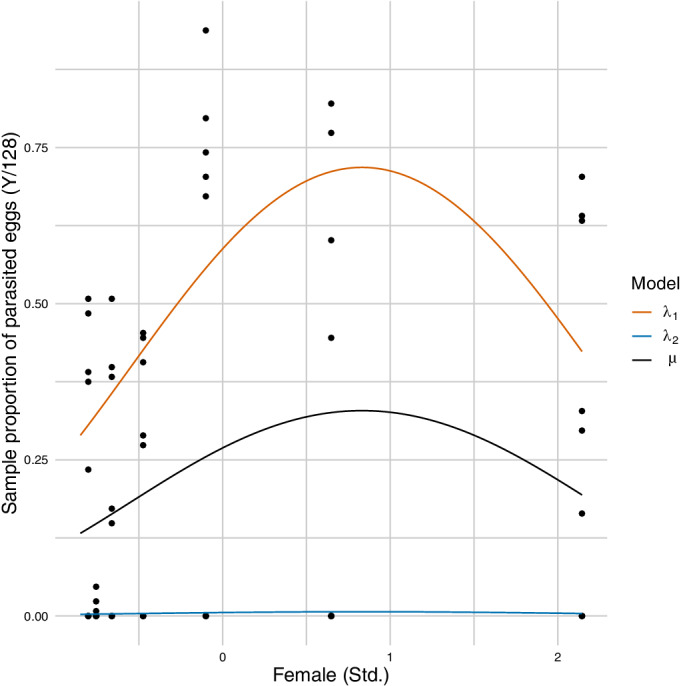
Bacteria data: Estimated FBBReg curves: $\lambda_{1}$ (orange), $\lambda_{2}$ (blue), and μ (black)[Color figure can be viewed at wileyonlinelibrary.com]

In particular, note that the FBBReg model exhibits a better fit (lower WAIC) and a higher precision (posterior mean of ϕ) than the BBReg model, which is the other model expressly developed to address overdispersion. This has direct consequences on the CS's of the regression coefficients, which are larger for the BBReg than for the FBBReg model. Moreover, the FBBReg model detects two well‐separated groups, as shown by the posterior means of p (0.453) and w (0.979). The group with the lower values of the response has posterior mean weight 1‐p equal to 0.547, which is very close to the proportion of zero values in the dataset, thus suggesting that the second component of the FBBReg model is dedicated to modeling the excess of zeros, as pointed out also by Figure 7. Due to the high proportion of zero values, we also fitted the ZIBinReg and ZIBBReg models. Both models detect the proportion of zeros quite accurately, though the FBBReg model exhibits a definitely better fit than the ZIBinReg model, and a comparable fit with respect to the ZIBBReg one.

Because of the experimental nature of the trials, Demétrio et al^21^ treat the number of females as a 7‐level factor. Therefore, for comparison purposes, we also fit the models using six dummy variables, with the level “2” as the baseline (see Table 2B). Once again, the FBBReg model exhibits the best fit (lowest WAIC) among non‐inflated models even more clearly. Here, the BinReg and the FBBReg coefficient estimates are similar, whereas those for BBReg are much lower, thus denoting a weaker effect of the covariate. Note that, also in this case, the higher precision estimates from the FBBReg model, compared with those of the BBReg model, result in narrower CS's for all regression coefficients.

Quite interestingly, for the BBReg model, the numeric covariate “female” is not significant (in Table 2A the CS's of $\beta_{1}$ and $\beta_{2}$ contain zero). Coherently, when the covariate “female” is treated as a factor, none of the six corresponding dummies is significant under the BBReg model, possibly because the precision is too low. Instead, the FBBReg model is able to distinguish between significant levels (namely, $\beta_{F4}$ with a negative impact with respect to the baseline, and $\beta_{F32}$ and $\beta_{F64}$ with a positive impact) and nonsignificant ones. Contrarily to its non‐inflated version, the ZIBBReg model displays a higher precision, so that it can detect some significant effects, but it performs only slightly better than the FBBReg model. Vice versa, the ZIBinReg model leads to an improvement with respect to its non‐inflated counterpart, but it performs worse than all the remaining models. Note that the two FBBReg models, obtained by treating the covariate as quantitative or as factor respectively, show coherence from the empirical/interpretative point of view, although they convey different kinds of information. This is shown by Figure S7 of the SM, which reports the estimated points corresponding to the categorized covariate. The only point showing a different behavior refers to the first dummy, which is due to the fact that the latter is characterized by a large number of zero values, and thus the corresponding point it is inevitably “attracted” downward.

Regardless of how “female” is treated, the posterior predictive checks (reported in the SM) show that the FBBReg model exhibits the best performance with respect to the overdispersion issue. Indeed, all the tools suggest that the BBReg model treats the extra variation (the variance posterior predictive *p*‐value is approximately equal to 0.5) at the expense of the modelization of the mean. Conversely, the FBBReg model's posterior predictive *p*‐values are all close to 0.5, the only exception being the one associated with the deviance, which, however, is the closest to 0.5 among those obtained from the considered non‐inflated models. All the above results suggest that the FBBReg can be the preferred model, as it performs as well as the ZIBBReg, even not being expressly developed to handle an excess of zeros, and better than all the remaining models.

**FIGURE 8:**
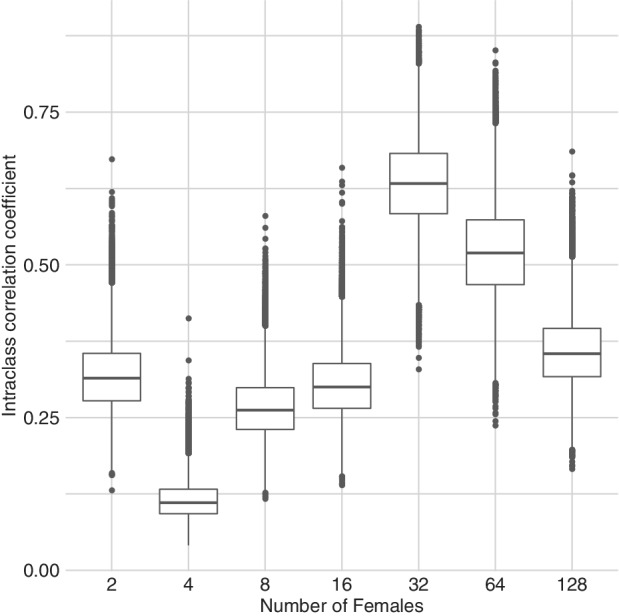
Bacteria data: ICC under the FBBReg model with the covariate “female” treated as a factor

A further plus of the FBBReg model is that it implies the modelization of the ICC as a function of the covariate (see Section 2.2), which allows deeper insight into the role and impact of the latter. More specifically, Figure 8 shows the simulated posterior distributions of the ICC for each level of the ordinal variable “female.” It clearly emerges that these distributions do depend on the level of “female,” especially for the significant levels $\beta_{F4}$, $\beta_{F32}$, and $\beta_{F64}$, and can assume large values, thus suggesting that the overdispersion can also be due to the correlated binary data forming the binomial counts.

### 5.2 Atomic bomb radiation data (latent groups)

We now consider an application based on the data by Otake and Prentice.^5^ For a large number of survivors of the atomic bombs in Hiroshima and Nagasaki, 100 cells have been analyzed, and the number Y of cells with chromosomal abnormalities has been recorded. Furthermore, for each subject, the estimated radiation exposure level (“dose”), expressed in rads, has been collected. Table 3A shows the posterior means and 95% CS's of the model parameters, as well as the WAIC values.

**TABLE 3:** Atomic bomb data: Posterior means and 95% CS's for the parameters

	(A) “Dose”		
Param.	BinReg	BBReg	FBBReg
$\beta_{0}$	−**4.130 (**−**4.184,** −**4.081)**	−**4.002 (**−**4.104,** −**3.898)**	−**4.122 (**−**4.223,** −**4.019)**
$\beta_{Dose}$	**0.0037 (0.0036, 0.0039)**	**0.0033 (0.0031, 0.0036)**	**0.0038 (0.0036, 0.0041)**
w	(–)	(–)	0.763 (0.609, 0.879)
ϕ	(–)	24.396 (21.071, 28.193)	32.975 (27.695, 38.958)
p	(–)	(–)	0.853 (0.745, 0.926)
WAIC	6163.2	4418.1	4378.6
	(B) “Dose” + “bomb”		
Param.	BinReg	BBReg	FBBReg
$\beta_{0}$	−**3.878 (**−**3.932,** −**3.824)**	−**3.818 (**−**3.918,** −**3.717)**	−**3.919 (**−**4.026,** −**3.815)**
$\beta_{Dose}$	**0.004 (0.004, 0.004)**	**0.003 (0.003, 0.004)**	**0.004 (0.004, 0.004)**
$\beta_{Bomb}$	−**0.849 (**−**0.932,** −**0.768)**	−**0.669 (**−**0.811,** −**0.531)**	−**0.705 (**−**0.865,** −**0.554)**
w	(–)	(–)	0.705 (0.461, 0.880)
ϕ	(–)	29.016 (24.954, 33.539)	39.543 (32.168, 50.729)
p	(–)	(–)	0.827 (0.536, 0.945)
WAIC	5698.9	4329.0	4297.1

*Note*: Regression coefficients in bold are related to 95% CS's not containing the zero value.

The effect of the dose of radiation exposure on the probability of chromosomal abnormalities is positive and significant for all three models, with the FBBReg showing the highest estimate (strongest effect). A graphical representation of the data together with the FBBReg regression curves can be found in Section 2.5 of the SM. The better performance of the FBBReg (ie, lower WAIC value) with respect to that of competitors is due to its ability to detect the two latent subpopulations forming the population under study (namely, Hiroshima and Nagasaki survivors). Indeed, the FBBReg model, thanks to its mixture structure, enables the determination of a “marginal” regression curve, which is a weighted mean of the clusters' regression curves. In particular, inspecting the regression curves, it clearly emerges that the one associated to $\lambda_{2}$ is dedicated to modeling Nagasaki survivors, whereas the curve for $\lambda_{1}$ models Hiroshima survivors. Based on the posterior predictive checks, the mean of the replicated outcomes agrees with the mean of the observed data under all three models. The panel on the variance discrepancy measure highlights that the BinReg model clearly suffers from the overdispersion problem since the distribution of the variance based on the replicated data is far away from that of the observed variance. However, both the BBReg and the FBBReg models handle the extra variation, with the posterior predictive *p*‐values showing the FBBReg model to be the preferred model.

The presence of two subpopulations is naturally better captured by a mixture model so to further compare the regression models in a more impartial scenario, we decided to include the city in which each subject survived the bomb (ie, the subpopulation) as a dummy covariate. Table 3B shows the parameter estimates and CS's, as well as the WAIC values for all the models. Quite interestingly, the FBBReg model still provides the best fit to the data. Further, note that the BinReg model is still affected by overdispersion issues, suggesting that the location groups are not the only source of extra variation. Awa et al^43^ suggest that important factors could be the age and type of aberration, meaning that further unobserved subpopulations could still exist. Indeed, even if the bomb dummy is included as a covariate, the FBBReg model still detects two groups of observations (posterior means of pandw equal to 0.827 and 0.705, respectively), showing no relevant differences between the posterior predictive checks without and with the location dummy.

### 5.3 Control mice data (outliers)

Preclinical studies represent an early step in the new drug development process. In particular, a potential drug must be tested on animals (eg, rabbits or mice) to establish if it can be safely administered to humans. In particular, some preclinical studies evaluate the undesired side effects of a new molecule in terms of negatively affecting the fertility of an animal by administering the drug to a male member of the considered species and mating it with one or more females. A greater number of deaths in fetal litters suggests a mutagenic effect. A control group is essential to assess whether there exists a drug‐associated adverse effect. Haseman and Soares^22^ report the number of fetal deaths in several control groups of mice for different litter sizes. Morgan^23^ analyzes the same data and declares that a mixture of a BB and a binomial distribution could provide a better fit than the standard binomial distribution since the binomial component of the mixture can accommodate outlying litters with high mortality. To evaluate the performance of our new model in the presence of outliers, we estimate the parameters of the BinReg, BBReg, and FBBReg models with no covariates (ie, $\text{logit}(\mu_{i})=\beta_{0},i=1,...,N)$ for only one control group proposed by Haseman and Soares,^22^ the CF1S group, which is composed by 524 litters. All three models provide similar estimates of the percentage μ of dead fetuses in the litters (see Table 4). However, the FBB model shows the best performance as it emerges from the WAIC values in Table 4, but also from the posterior predictive *p*‐values. Indeed, the binomial and BB distributions are clearly affected by the overdispersion problem, as shown by the variance posterior *p*‐values, which are far from 0.5.

**TABLE 4:** Control mice data: Posterior means and 95% CS's for the parameters

Param.	BinReg	BBReg	FBBReg
$\beta_{0}$	−**2.323 (**−**2.407,** −**2.241)**	−**2.310 (**−**2.423,** −**2.199)**	−**2.304 (**−**2.422,** −**2.183)**
μ	0.089 (0.083, 0.096)	0.09 (0.081, 0.1)	0.091 (0.082, 0.101)
w	(–)	(–)	0.659 (0.207, 0.983)
ϕ	(–)	13.667 (10.062, 18.432)	18.490 (12.184, 29.067)
p	(–)	(–)	0.031 (0.001, 0.388)
WAIC	1688.3	1560.5	1555.5

*Note*: Regression coefficients in bold are related to 95% CS's not containing the zero value.

**FIGURE 9:**
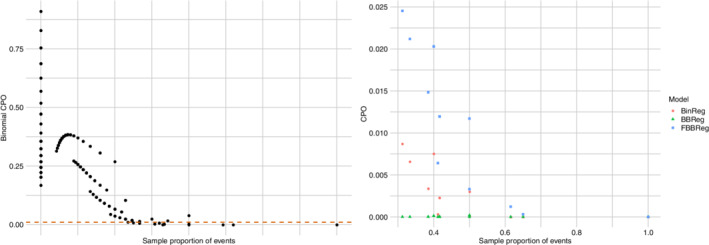
Control mice data. Left panel: Binomial $\hat{\text{CPO}}$'s for the CF1S data, horizontal dashed line representing a threshold of 0.01. Right panel: $\hat{\text{CPO}}$'s under the BinReg, BBReg, and FBB models for the litters with binomial CPO ≤ 0.01[Color figure can be viewed at wileyonlinelibrary.com]

Since the literature reports that these data are affected by outliers, the overdispersion can be plausibly ascribed to their presence. Therefore, we further compare the three models using the CPO measure. The left panel in Figure 9 shows the $\hat{\text{CPO}}$ values under the binomial model. Although a large number of data points are modeled well by the binomial distribution, there is a group of litters characterized by high mortality and a low CPO value. We focus on the 16 litters with a binomial $\hat{\text{CPO}}$ ≤0.01 and compare their $\hat{\text{CPO}}$ value in the three considered models in the right panel of Figure 9. The FBB model exhibits the highest $\hat{\text{CPO}}$ values for all the outlying litters as it dedicates a mixture component to them. Since outliers are characterized by high mortality, and because of the constraint $\lambda_{1}>\lambda_{2}$, the mixture component that models the outliers is the first one. This is also confirmed by the posterior mean of *p* = 0.031 ≈ 16/524. Finally, note that the BB model exhibits the worst CPO performance, thus confirming its inadequacy in the presence of outliers.

## 6 CONCLUDING REMARKS

The overdispersion issue, often affecting the BinReg model, is usually addressed by the well‐known BBReg model. However, the latter does not always succeed in handling multiple concomitant sources of extra variability. In this study, we proposed a new mixture distribution for constrained counts, and a novel regression model based on it, namely, the FBBReg model. This model involves a set of parameters that have a clear interpretation in terms of (possible) latent subpopulations. Indeed, results from an extensive simulation study and from some applications to real biomedical datasets show that this model can handle the extra variation due to a missing covariate (latent groups) and, surprisingly, it can also easily adapt to some other important sources of overdispersion that practitioners commonly encounter, namely, outliers and/or an excess of zero observations. The model achieves this by automatically dedicating a mixture component to them. The estimation issues are addressed using a Bayesian approach which can be easily implemented through standard tools such as Stan. In particular, posterior predictive checks (plots and posterior predictive *p*‐values) prove to be a powerful tool for detecting overdispersion in a Bayesian context. Moreover, they can also be easily interpreted by practitioners. Indeed, in our context, they provide the important result that not only the BinReg model, but also the BBReg model is often inadequate for handling the extra variation, whereas the FBBReg model produces a very good, sometimes outstanding, performance in many (simulated and real) applications.

Due to the promising features of the FBBReg model, in future work, we plan to extend it in at least three directions. A first relevant extension is the inclusion of random effects to allow for responses with a hierarchical structure (typically measured longitudinally or clustered), so that within‐subject correlation can be handled. Moreover, since the parameters ϕ,p,andw deserve a clear interpretation, it seems worthwhile to explore the possibility of letting some of them depend on covariates too. This could greatly increase the flexibility of the model, enabling it to better fit and interpret more complex data patterns. Moreover, the presence of two groups of outliers (above and below the main cloud as in the simulative scenario of Section 4.3) could be handled in a parsimonious way (only one parameter more) via inflation.

Finally, we plan to work on the multivariate version of the FBBReg, which can be obtained by compounding the multinomial distribution with the multivariate FB distribution, that is, the FD. This could broaden the number of latent subpopulations and possibly lead to an extension of the Dirichlet‐multinomial model,^44^, ^45^ allowing us to overcome some of its drawbacks such as its unimodality and the stiffness of its dependence structure.

## CONFLICT OF INTEREST

The authors declare no potential conflict of interest.

## Supporting information

**Data S1:** Supporting material

## Data Availability

The data that support the findings of this study are available from the corresponding author upon reasonable request.
