# Supplementary material for: A new regression model for overdispersed binomial data accounting for outliers and an excess of zeros
Source: Stat Med. 2021 May 7;40(17):3895–914. doi: 10.1002/sim.9005 (PMC8360060; doi:10.1002/sim.9005)
Supplement: Supplementary file 1 — Data S1: Supporting material [file SIM-40-3895-s001.pdf]

Supplementary Material for "A new regression model for overdispersed binomial data accounting for outliers and an excess of zeros"

by R. Ascari and S. Migliorati

April 7, 2021

1 Robustness with respect to prior elicitation

In this section we perform a sensitivity study to investigate whether different choices of the priors affect the posterior distributions. More precisely, we consider one replication for each of the scenarios described in Section 4.1 of the main paper and we focus on different choices of the prior and/or hyperparameters of the regression coefficients $\boldsymbol{\beta}$ and the precision parameter ϕ.

In particular, we select a diffuse multivariate normal distribution for $\boldsymbol{\beta}$, as described in the paper, and we choose four different values of the prior standard deviation to account for different amounts of vagueness: $\sqrt{10}$, 10, 50, and 100. Regarding the precision parameter ϕ, we consider both a Gamma(kg, g), with $g = 0.001$ to induce a large variance around the prior mean k ($k \in \{1, 10, 50\}$), and a uniform prior on $\theta = 1/(1 + \phi)$. Tables S1 and S2 show the posterior means and credible sets of the involved parameters as well as the WAIC values based on four chains of length 30000 each, whereas Figures S1 and S2 show the posterior density of each parameter. All the numerical and graphical summaries do not significantly depend on the prior/hyperparameter we select, pointing a robustness of the estimation procedure.

Table S1: Posterior means and credible sets for parameters β_0 and β_1 in the four considered scenarios under different choices of the prior standard deviation.

		β_0		β_1		
Scenario	Prior SD	Post. Mean	CS	Post. Mean	CS	WAIC
(1)	$\sqrt{10}$	1.0157	(0.9544, 1.0773)	2.9466	(2.8172, 3.0702)	1066.2
	10	1.0157	(0.9545, 1.0782)	2.9470	(2.8190, 3.0708)	1066.2
	50	1.0157	(0.9552, 1.0780)	2.9475	(2.8197, 3.0715)	1066.3
	100	1.0156	(0.9541, 1.0780)	2.9464	(2.8182, 3.0703)	1066.3
(2)	$\sqrt{10}$	1.0810	(0.7999, 1.3642)	2.9793	(2.4248, 3.5387)	1056.7
	10	1.0857	(0.8051, 1.3675)	2.9952	(2.4433, 3.5563)	1056.7
	50	1.0881	(0.8074, 1.3685)	3.0025	(2.4440, 3.5582)	1056.7
	100	1.0874	(0.8116, 1.3652)	3.0027	(2.4529, 3.5569)	1056.5
(3)	$\sqrt{10}$	1.1088	(0.9403, 1.2769)	2.9323	(2.6842, 3.1829)	1252.8
	10	1.1094	(0.9426, 1.2756)	2.9361	(2.6881, 3.1855)	1252.8
	50	1.1101	(0.9432, 1.2778)	2.9365	(2.6865, 3.1838)	1252.7
	100	1.1112	(0.9450, 1.2797)	2.9383	(2.6894, 3.1882)	1252.7
(4)	$\sqrt{10}$	1.1245	(0.8654, 1.3537)	2.6632	(2.2689, 3.0380)	1213.2
	10	1.1335	(0.8716, 1.3615)	2.6791	(2.2795, 3.0528)	1213.3
	50	1.1333	(0.8706, 1.3587)	2.6779	(2.2819, 3.0522)	1213.3
	100	1.1323	(0.8677, 1.3612)	2.6769	(2.2763, 3.0585)	1213.4

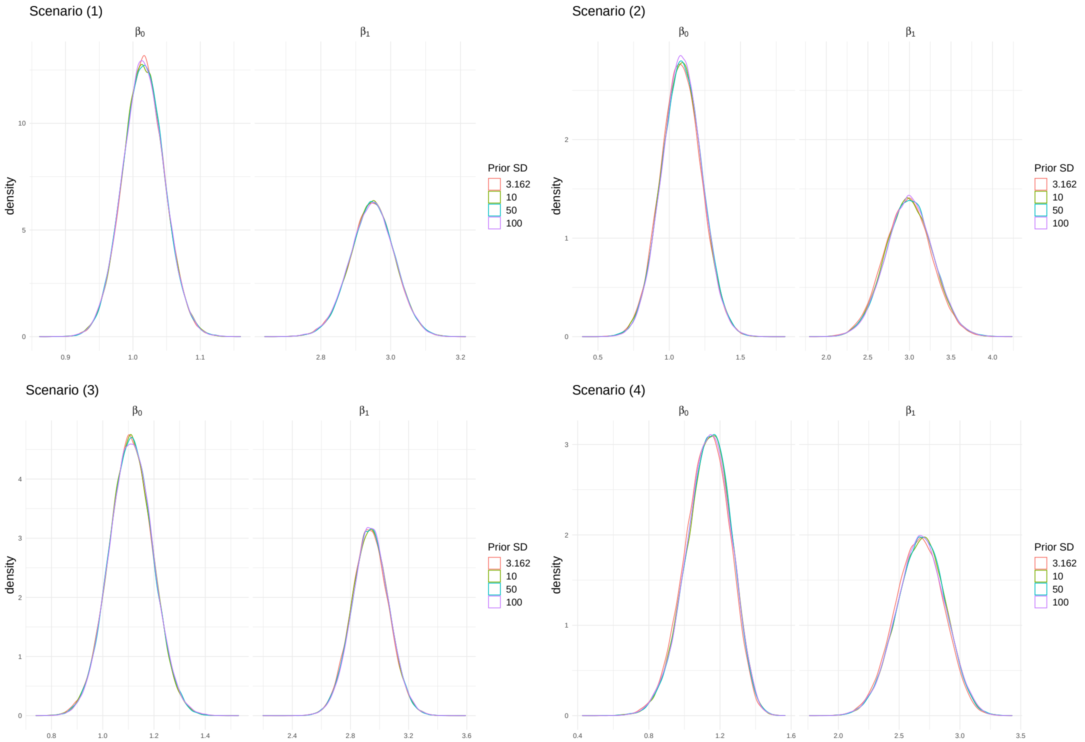

Figure S1: Posterior densities of parameters β_0 and β_1 in the four considered scenarios under different choices of the prior standard deviation.

Table S2: Posterior means and credible sets for parameters ϕ and θ in the four considered scenarios under different choices of the prior.

		ϕ		θ		
Scenario	Prior	Post. Mean	CS	Post. Mean	CS	WAIC
(1)	$k = 1$	104.5696	(70.4298, 155.2766)	0.0099	(0.0064, 0.0140)	1066.3
	$k = 10$	104.3997	(70.3815, 154.0412)	0.0099	(0.0064, 0.0140)	1066.4
	$k = 50$	104.5963	(70.1162, 154.6629)	0.0099	(0.0064, 0.0141)	1066.4
	Uniform on θ	100.6444	(68.1239, 147.6202)	0.0102	(0.0067, 0.0145)	1066.2
(2)	$k = 1$	1.869	(0.936, 3.2920)	0.3658	(0.2330, 0.5165)	1056.6
	$k = 10$	1.8549	(0.9348, 3.3026)	0.3675	(0.2324, 0.5168)	1056.6
	$k = 50$	1.8680	(0.9382, 3.3311)	0.3662	(0.2309, 0.5159)	1056.7
	Uniform on θ	1.8039	(0.9299, 3.2265)	0.3735	(0.2366, 0.5181)	1056.7
(3)	$k = 1$	19.1915	(13.0939, 26.3521)	0.0510	(0.0366, 0.0710)	1252.6
	$k = 10$	19.1821	(13.1364, 26.3187)	0.0510	(0.0366, 0.0707)	1252.7
	$k = 50$	19.2399	(13.1731, 26.4118)	0.0508	(0.0365, 0.0706)	1252.7
	Uniform on θ	18.6596	(12.7343, 25.7002)	0.0523	(0.0375, 0.0728)	1252.7
(4)	$k = 1$	16.5222	(11.6696, 22.4500)	0.0585	(0.0426, 0.0789)	1213.4
	$k = 10$	16.4761	(11.620, 22.3298)	0.0586	(0.0429, 0.0792)	1213.4
	$k = 50$	16.5100	(11.6710, 22.3669)	0.0585	(0.0428, 0.0789)	1213.4
	Uniform on θ	16.1093	(11.3560, 21.9325)	0.0599	(0.0436, 0.0809)	1213.4

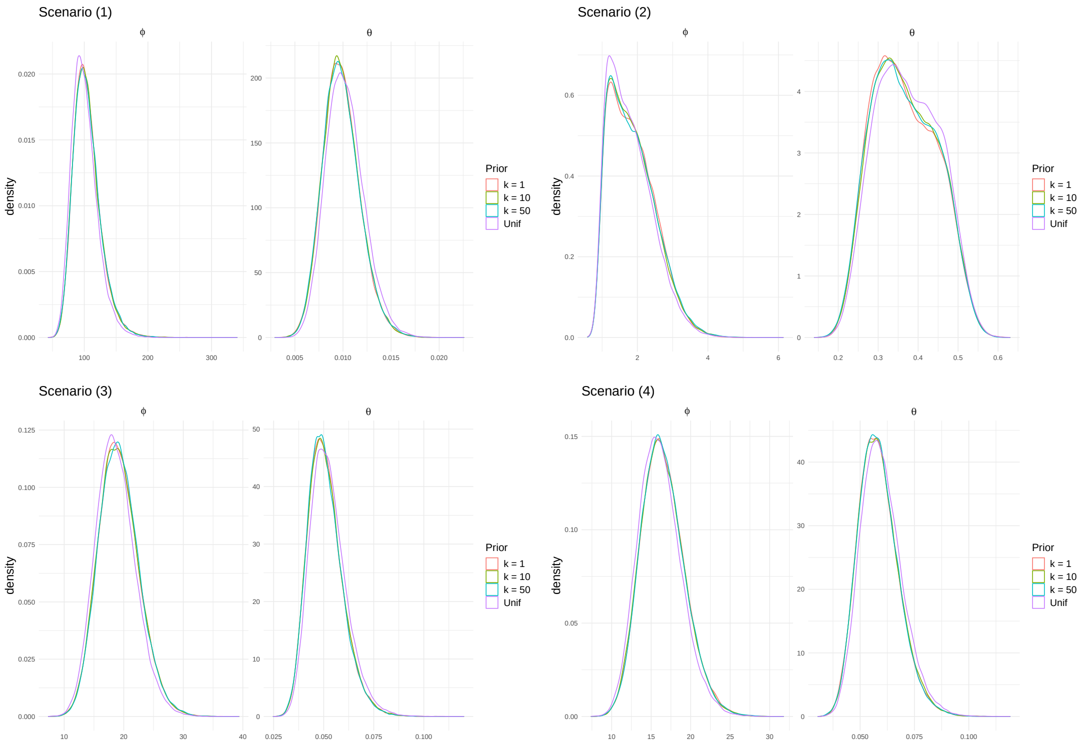

Figure S2: Posterior densities of parameters ϕ and θ in the four considered scenarios under different choices of the prior.

2 Additional Figures

2.1 Intraclass correlation coefficient

A clear picture of the behavior of the FBB's intraclass correlation coefficient ρ_{FBB} (see formula (14) of the main paper) is given in Figure S3, which presents some iso-correlation curves for different values of w and p. It shows that, for fixed ϕ, w, and μ, the value of p that maximizes ρ_{FBB} is $p = \mu$. Moreover, it highlights that the ICC can take quite large values (even near 1).

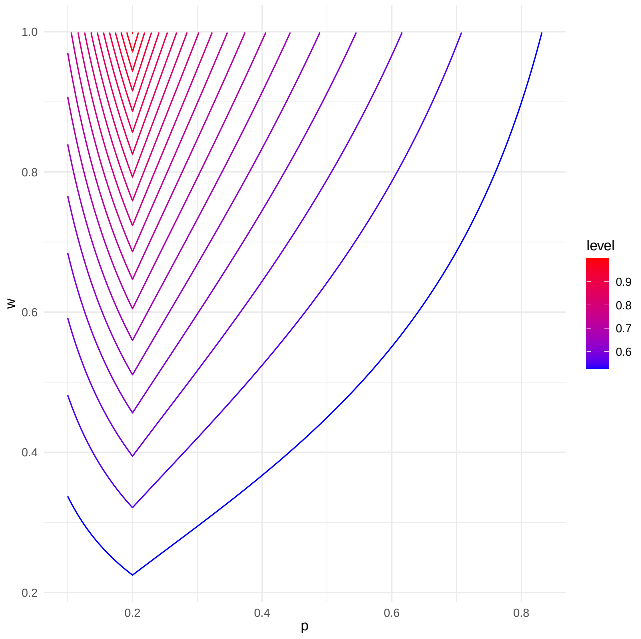

Figure S3: Intraclass correlation coefficient ρ_{FBB} as a function of p and w, with $\mu = 0.2$ and $\phi = 1$.

2.2 Probability of zeros

Figures S4 illustrates the behavior of the probability $\delta_0^{FBB(\mu,w,\phi,p)}$ of the event “zero successes among n trials” under the FBB model (see formula (16) of the main paper) as some parameters vary. Moreover, Figure S5 shows its contour plots as a function of μ and ϕ for fixed p and w.

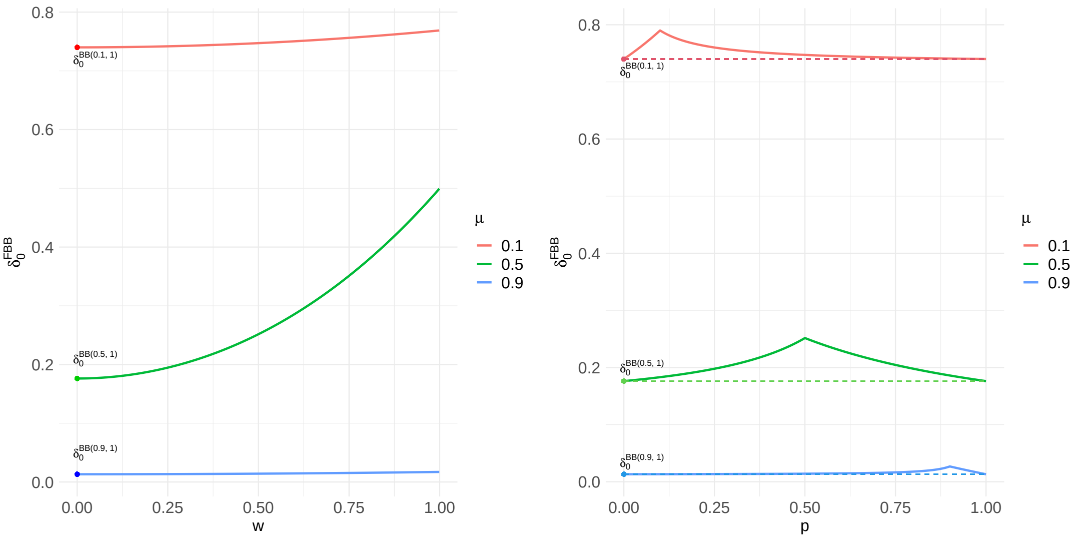

Figure S4: Left panel: probability of zero values under the FBB model as a function of w for fixed $p = 0.5$ and $\phi = 1$, and different values of μ. Right panel: probability of zero values as a function of μ and ϕ for fixed $n = 10$, $p = 0.3$, and $w = 0.2$.

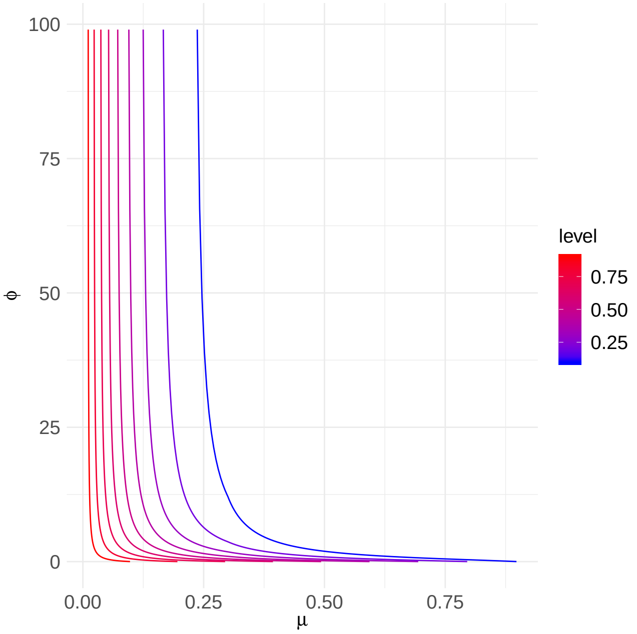

Figure S5: Probability of zero values under the FBB model as a function of μ and ϕ for fixed $n = 10$, $p = 0.3$, and $w = 0.2$.

2.3 Model fit simulation study (Section 4.1): one replication from Scenario (4)

In Section 4.1 of the main paper (scenario (4)), we used a data generating process based on a generic mixture of two BBReg's. One component of the mixture is characterized by a very small precision parameter. In Figure S6 we show a randomly selected replication from this scenario. It is easy to see the two components, as well as the excess of zeros due to the low precision of the second component.

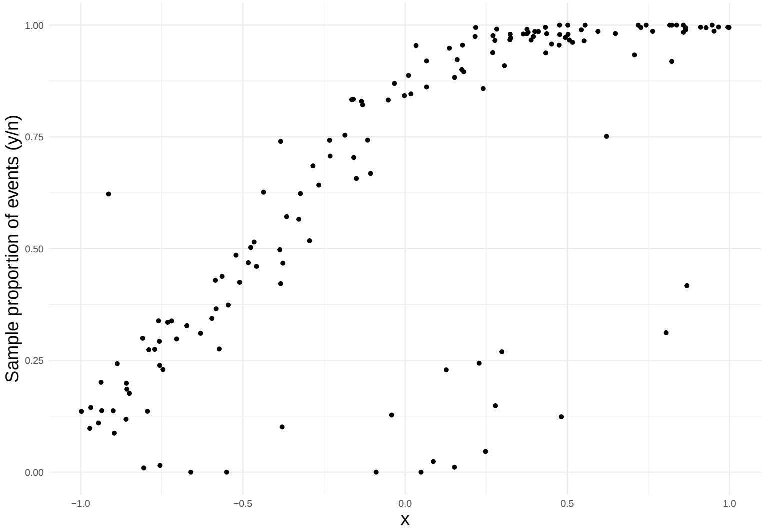

Figure S6: Model fit simulation study of Section 4.1, scenario (4): one randomly selected replication.

2.4 Bacteria data (Section 5.1)

In Section 5.1 of the main paper, we considered two regression models for the bacteria data, treating the covariate "number of females" as quantitative (model (a)) and as a factor (model (b)). In Figure S7 we compare the estimated means for the two models, showing that they provide coherent estimates for most of the observed values of the covariate.

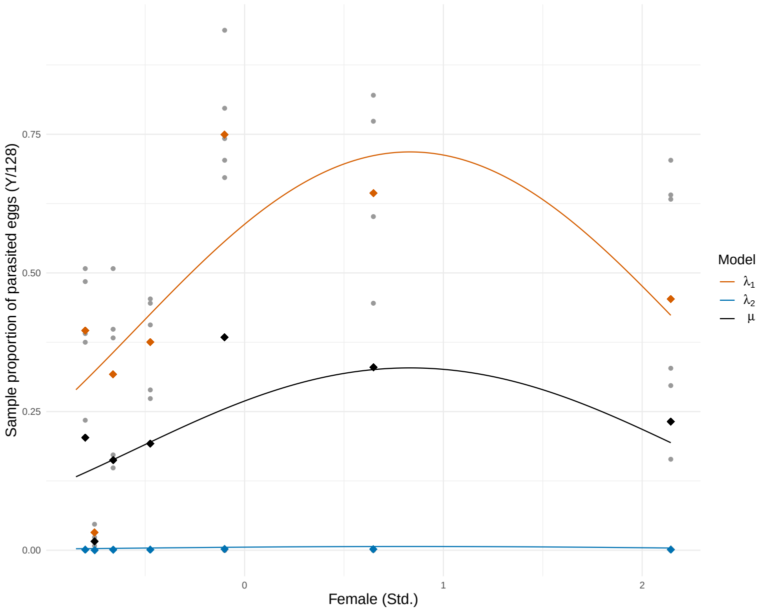

Figure S7: Bacteria data: continuous curves refer to FBBReg regression curves for model (a), whereas diamond points refer to FBBReg means for model (b).

2.5 Atomic Bombs data (Section 5.2)

Data used in Section 5.2 are plotted as points in Figure S8, where the "dose" variable (x-axis) has been jittered for a better visualization. Each subject is represented by a point colored according to the city in which she/he survived the bomb.

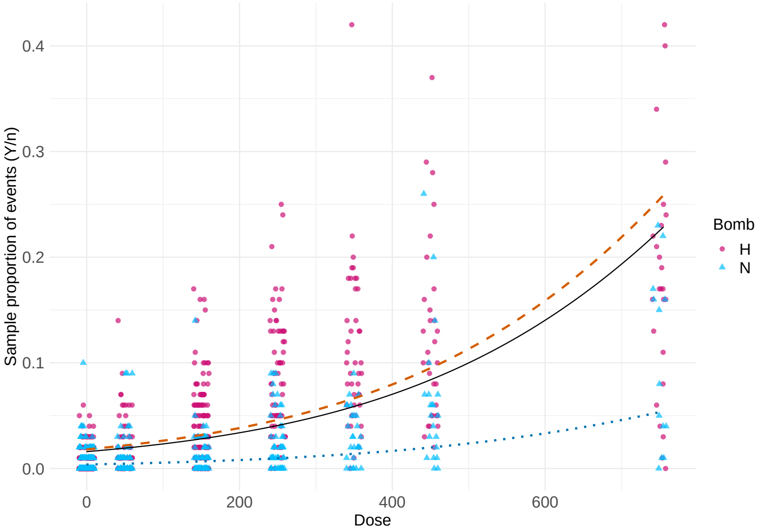

Figure S8: Atomic bomb data: purple circles represent Hiroshima observations, and light-blue triangles represent Nagasaki ones. The black solid line represents the FBBReg curve μ whereas orange dashed and blue dotted lines represent the group regressions λ_1 and λ_2, respectively.

3 Additional Tables

3.1 Excess of zeros simulation study (Section 4.2)

Table S3 reports some details on posterior distribution of p from the "excess of zeros" simulation study.

Table S3: Excess of zeros simulation study: posterior means, standard deviations and 95% credible sets (CS) for parameter p.

Scenario	Post. Mean	Post. SD	95 % CS
5% of zeros	0.9905	0.0067	(0.9845, 0.9945)
10% of zeros	0.9790	0.0222	(0.9072, 0.9913)
20% of zeros	0.7822	0.0742	(0.6437, 0.9375)
50% of zeros	0.4684	0.0273	(0.4157, 0.5187)

3.2 Outlier contamination simulation study (Section 4.3)

Table S4 reports some details on posterior distribution of p from the "outlier contamination" simulation study.

Table S4: Outlier contamination simulation study: posterior means, standard deviations and 95% credible sets (CS) for parameter p.

Scenario	Post. Mean	Post. SD	95 % CS
(I)	0.0582	0.0299	(0.0153, 0.1280)
(II)	0.9928	0.0032	(0.9855, 0.9978)
(III)	0.9810	0.0095	(0.9578, 0.9945)

4 Posterior predictive checks and p-values

4.1 Bacteria data (Section 5.1)

Table S5: Bacteria data: estimated posterior predictive p-values.

	(a) “number of female” as quantitative			(b) “number of female” as factor		
	Mean	Variance	Deviance	Mean	Variance	Deviance
BinReg	0.5018	0	0	0.5049	0	0
BBReg	0.1326	0.4812	0.0157	0.1469	0.5166	0.0151
FBBReg	0.4386	0.4574	0.6142	0.5454	0.5563	0.6988
ZIBinReg	0.4986	0.0172	0	0.4380	0.1794	0
ZIBBReg	0.4449	0.5654	0.3509	0.5437	0.6009	0.4622

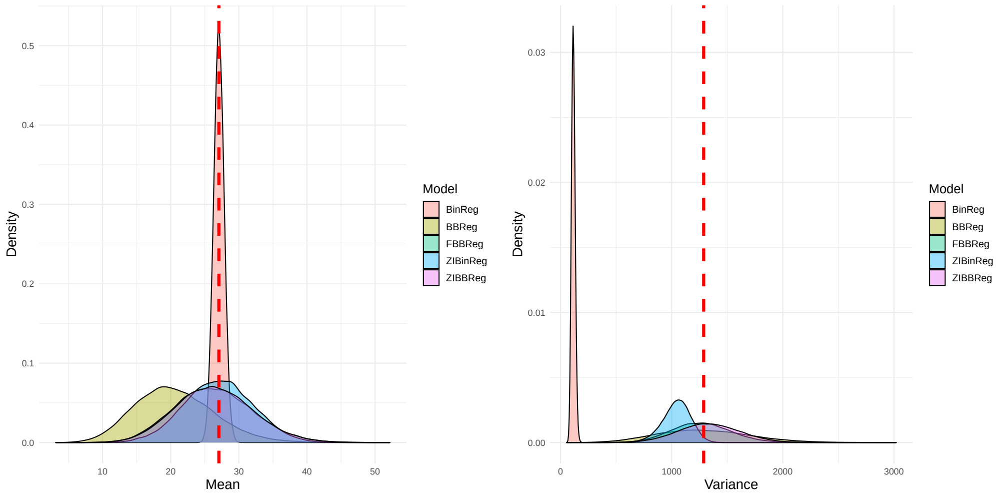

Figure S9: Bacteria data: posterior predictive checks with the covariate “number of female” treated as numeric.

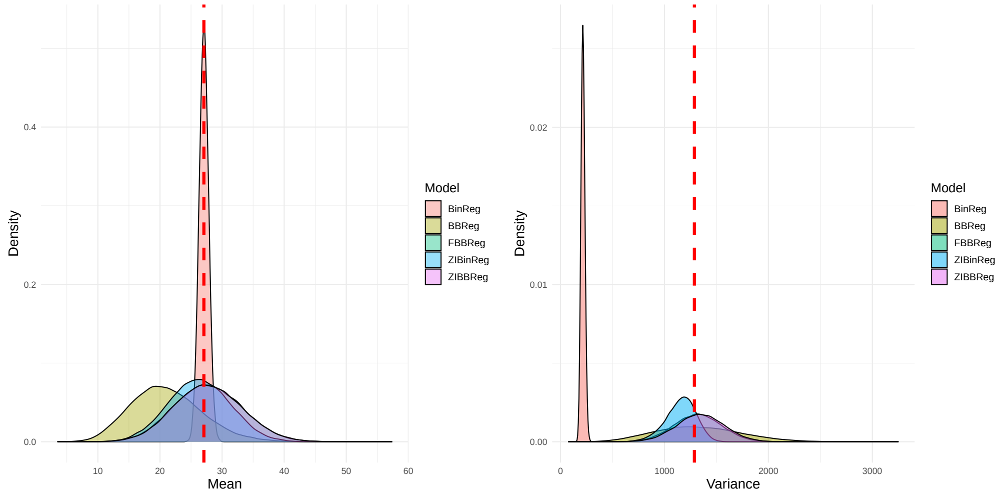

Figure S10: Bacteria data: posterior predictive checks with the covariate "number of female" treated as a factor.

4.2 Atomic bomb radiation data (Section 5.2)

Table S6: Atomic Bombs: estimated posterior predictive p-values.

	(a) "dose"			(b) "dose" + "bomb"		
	Mean	Variance	Deviance	Mean	Variance	Deviance
BinReg	0.5003	0	0	0.4936	0	0
BBReg	0.6075	0.1395	0.7779	0.6028	0.1697	0.7835
FBBReg	0.7829	0.7161	0.7300	0.7747	0.7833	0.7470

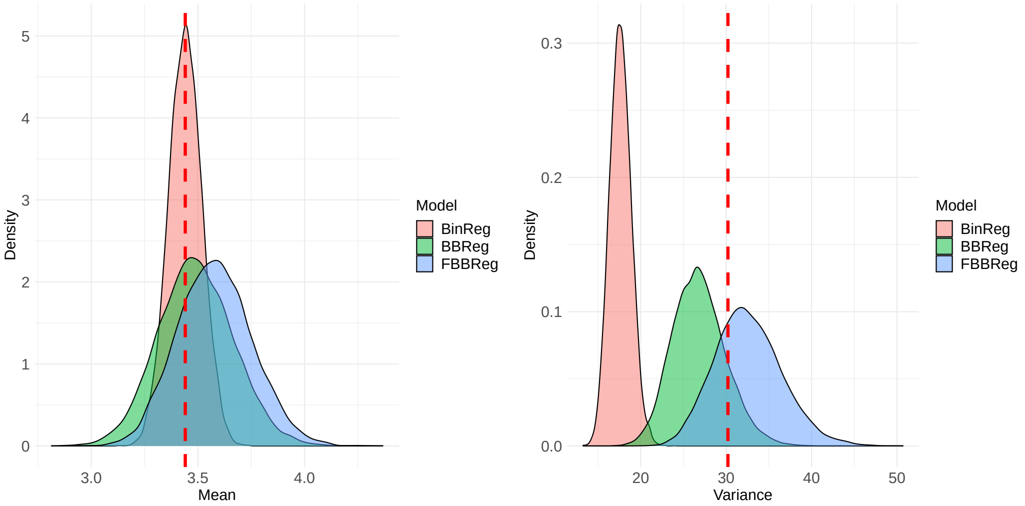

Figure S11: Atomic bomb data with one covariate ("dose"): posterior predictive checks.

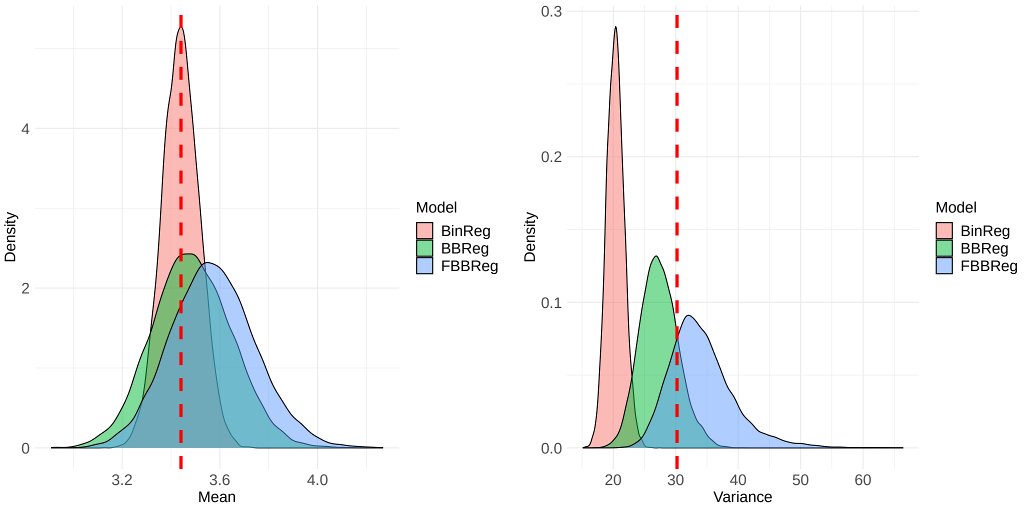

Figure S12: Atomic bomb data with two covariates ("dose" and "bomb"): posterior predictive checks.

4.3 Control mice data (Section 5.3)

Table S7: Control mice data: estimated posterior predictive p-values.

	Mean	Variance	Deviance
BinReg	0.5074	0	0
BBReg	0.5633	0.2965	0.5782
FBBReg	0.5851	0.5269	0.5538

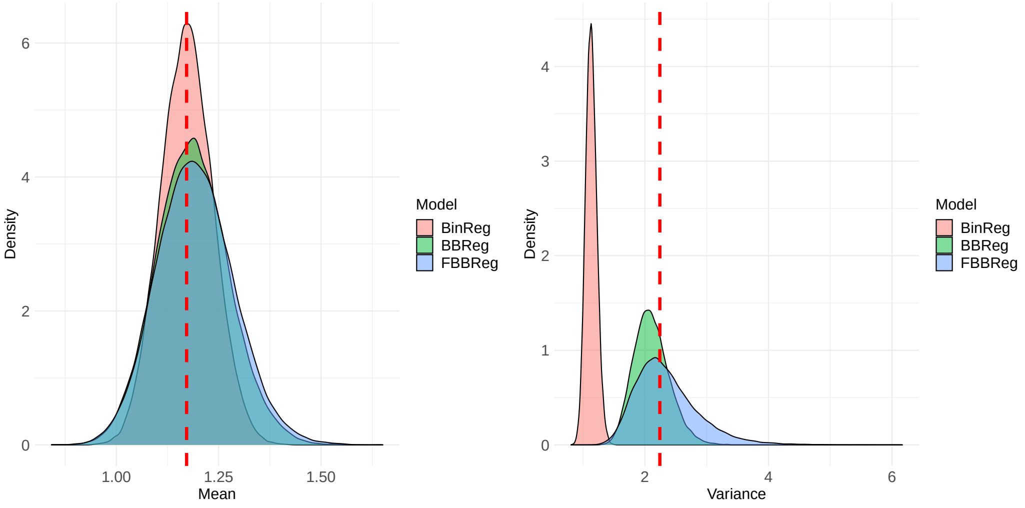

Figure S13: Control mice data: posterior predictive checks.
